# Immune Reconstitution of Patients Who Recovered From Steroid-Refractory Acute Graft-Versus-Host Disease After Basiliximab Treatment

**DOI:** 10.3389/fonc.2022.916442

**Published:** 2022-07-15

**Authors:** Dao-Xing Deng, Shuang Fan, Xiao-Hui Zhang, Lan-Ping Xu, Yu Wang, Chen-Hua Yan, Huan Chen, Yu-Hong Chen, Wei Han, Feng-Rong Wang, Jing-Zhi Wang, Xu-Ying Pei, Ying-Jun Chang, Kai-Yan Liu, Xiao-Jun Huang, Xiao-Dong Mo

**Affiliations:** ^1^ Peking University People’s Hospital, Peking University Institute of Hematology, National Clinical Research Center for Hematologic Disease, Beijing Key Laboratory of Hematopoietic Stem Cell Transplantation, Beijing, China; ^2^ Peking-Tsinghua Center for Life Sciences, Academy for Advanced Interdisciplinary Studies, Peking University, Beijing, China; ^3^ Research Unit of Key Technique for Diagnosis and Treatments of Hematologic Malignancies, Chinese Academy of Medical Sciences, Beijing, China

**Keywords:** Immune reconstitution, basiliximab, steroid-refractory, acute graft-versus-host disease, haploidentical, allogeneic hematopoietic stem cell transplantation

## Abstract

We aimed to identify the characteristics of immune reconstitution (IR) in patients who recovered from steroid-refractory acute graft-versus-host disease (SR-aGVHD) after basiliximab treatment. A total of 179, 124, 80, and 92 patients were included in the analysis for IR at 3, 6, 9, and 12 months, respectively, after haploidentical donor hematopoietic stem cell transplantation (HID HSCT). We observed that IR was fastest for monocytes and CD8^+^ T cells, followed by lymphocytes, CD3^+^ T cells, and CD19^+^ B cells and slowest for CD4^+^ T cells. Almost all immune cell subsets recovered comparably between patients receiving <5 doses and ≥5 doses of basiliximab. Most immune cell subsets recovered comparably between SR-aGVHD patients who recovered after basiliximab treatment and event-free HID HSCT recipients. Patients who recovered from SR-aGVHD after basiliximab treatment experienced satisfactory IR, which suggested that basiliximab may not have prolonged the negative impact on IR in these patients.

## Introduction

Human leukocyte antigen (HLA) haploidentical donor (HID) has become one of the most important donors for allogeneic hematopoietic stem cell transplantation (allo-HSCT) (1), which accounts for the proportion at 60% among all of the allo-HSCTs in China (2). However, although many strategies [e.g., anti-thymocyte globulin (ATG)] are used to prevent acute graft-versus-host disease (aGVHD), there are still 40%–50% of patients suffering from grade II–IV aGVHD, which remains one of the major causes of early mortality after HID HSCT (3).

Corticosteroids are the first-line treatment for aGVHD, but their response rate can only reach nearly 50% (4, 5). In addition, Liu et al. (6) reported that the complete response (CR) rate of steroid treatment for grade II–IV aGVHD was only 34.1% in HID HSCT recipients. Thus, steroid-refractory (SR)-aGVHD is common; however, there is no consensus on the best therapeutic options for HID HSCT recipients (7). Considering that interleukin-2 (IL-2) plays a critical role in aGVHD pathogenesis, IL-2 receptor blockade (e.g., basiliximab) is important for SR-aGVHD therapy (8–10). Several studies have identified the efficacy of basiliximab in SR-aGVHD patients (11–14). In addition, 2 cohorts with large samples of HID HSCT recipients reported that the overall response rate (ORR) of basiliximab therapy was approximately 80% in SR-aGVHD patients and the long-term survival could achieve more than 60% (15, 16). Thus far, basiliximab has been the most important treatment for SR-aGVHD in China (17).

Immune reconstitution (IR) was important for long-term survivors after allo-HSCT, and patients with poor IR were associated with a higher risk of infections and non-relapse mortality (NRM) (18, 19). Active aGVHD could influence IR (20). In addition, SR-aGVHD patients would receive intensive immunosuppressants, which might show a prolonged negative impact on IR even in those who recovered after therapies. Because more and more SR-aGVHD patients could be cured and achieve long-term survival after second-line or third-line therapies, it was necessary to identify the characteristics of IR in these patients. However, to the best of our knowledge, no study had identified the characteristics of IR in patients who recovered from SR-aGVHD and achieved GVHD-free survival.

Particularly, SR-aGVHD mostly occurs in HID HSCT recipients (15). Although some authors reported the characteristics of IR in event-free HID HSCT recipients (21, 22), patients with aGVHD were excluded and whether patients who recovered from SR-aGVHD had the same characteristics of IR was unclear.

Thus, we aimed to identify the characteristics of IR in SR-aGVHD patients who recovered from SR-aGVHD with the help of basiliximab treatment after HID HSCT.

## Methods

### Patients

**Figure 1:**
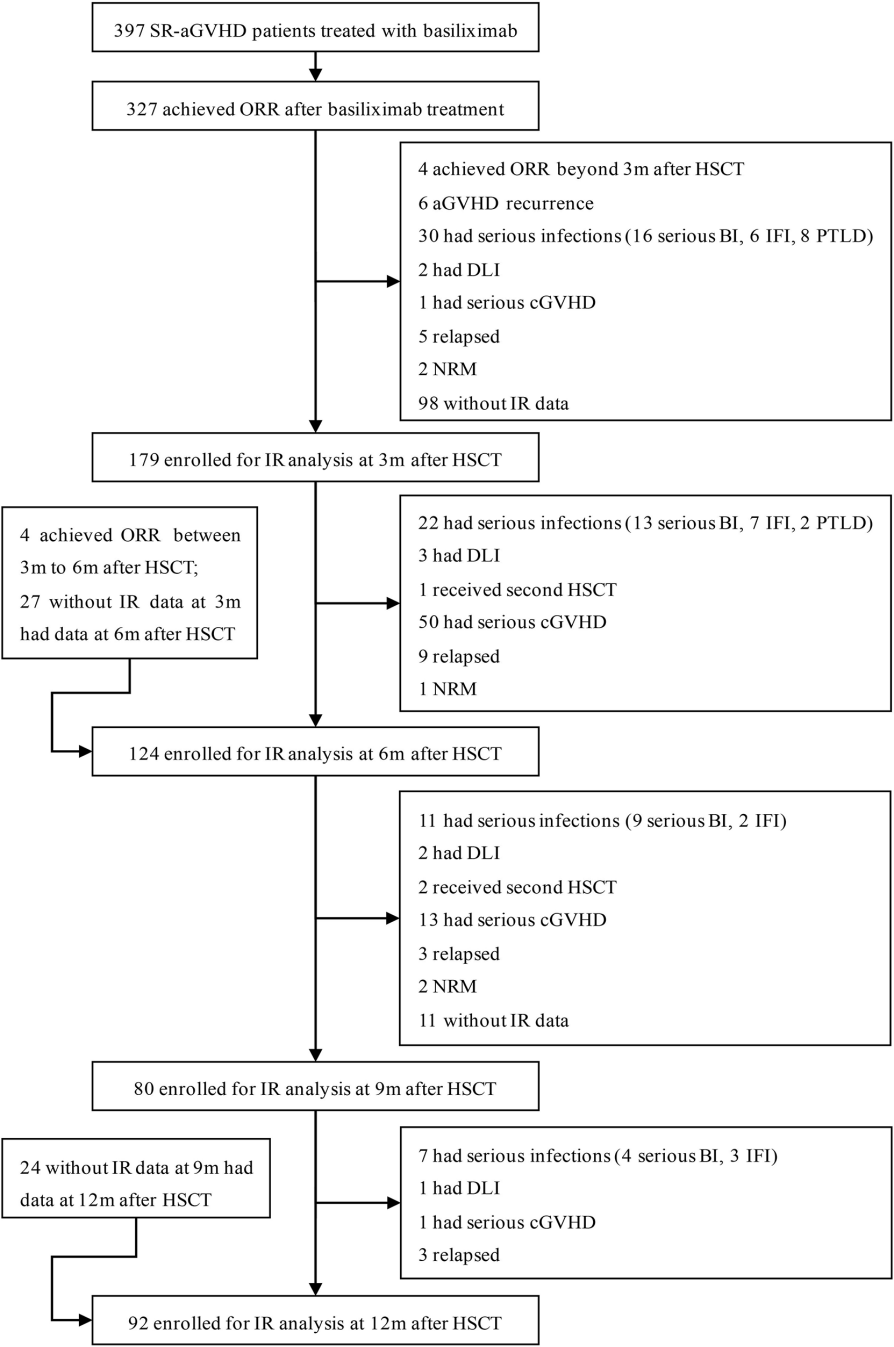
Diagram of patients enrolled. A total of 327 SR-aGVHD patients who showed an ORR to basiliximab treatment were eligible, and 179, 124, 80, and 92 patients were included in the final analysis for IR at 3, 6, 9, and 12 months, respectively, after HID HSCT. HID HSCT, haploidentical donor hematopoietic stem cell transplantation; IR, immune reconstitution; NRM, non-relapse mortality; ORR, overall response rate; SR-aGVHD, steroid-refractory acute graft-versus-host disease.

Consecutive HID HSCT recipients who recovered from SR-GVHD after basiliximab therapy at the Peking University Institute of Hematology (PUIH) from January 2016 to December 2018 were enrolled in this retrospective study. In order to reflect the true rule of IR in patients who recovered from SR-aGVHD after basiliximab, we excluded patients who did not achieve an ORR after basiliximab treatment. In addition, patients who had other situations that could influence IR, including aGVHD recurrence, serious infection [i.e., serious bacterial infections, invasive fungal infection (IFI), and posttransplant lymphoproliferative disorders (PTLDs)], moderate to severe chronic GVHD (cGVHD), relapse, NRM, receiving donor lymphocyte infusion (DLI), receiving second allo-HSCT, were all excluded. Patients who did not monitor IR after HSCT were also excluded (**Figure 1**). Considering that most of the patients recovered from SR-aGVHD within 3 months after HSCT, we focused on the data of IR at 3, 6, 9, and 12 months following HID HSCT. The last follow-up for survivors was conducted on September 30, 2021. To further identify the IR in patients who recovered from SR-aGVHD, two historical cohorts (event-free HID HSCT recipients: n = 85 and healthy donors: n = 41) reported by Pei et al. (21) were also included as control. The study protocol was approved by the institutional review board of Peking University People’s Hospital and was conducted in accordance with the Declaration of Helsinki.

### Transplant Regimen

For patients with hematologic malignancies, the preconditioning regimen mainly included cytarabine, busulfex, cyclophosphamide, simustine, and rabbit anti-thymocyte globulin (ATG) (23, 24). For patients with severe aplastic anemia, the preconditioning regimen mainly included busulfex, cyclophosphamide, and ATG (25). We used cyclosporine A (CSA), mycophenolate mofetil (MMF), and methotrexate (MTX) to prevent GVHD (26, 27). The protocol of basiliximab (Simulect; Novartis Pharma AG, Basel, Switzerland) treatment was shown in the Supplementary methods (15, 28).

### Immune Reconstitution Analysis

The total lymphocyte and monocyte counts were detected by peripheral blood cell analysis at 1, 2, 3, 6, 9, and 12 months following HID HSCT. Immune cell subsets were recognized and measured by multiparameter flow cytometry (MFC) at 1, 2, 3, 6, 9, and 12 months following allo-HSCT, as described previously (Supplementary methods) (22). Serum IgA, IgG, and IgM levels were detected by immunonephelometry.

### Definition

Good IR was defined as at least 3 types of major immune cells (i.e., monocytes, lymphocytes, CD19^+^ B cells, CD3^+^ T cells, CD4^+^ T cells, and CD8^+^ T cells) meeting or exceeding the median value of event-free HID HSCT cohort. cGVHD was diagnosed and categorized according to accepted international criteria (29). The definition of relapse, NRM, disease-free survival (DFS), and overall survival (OS) were shown in the Supplementary methods.

### Statistical Analysis

Continuous variables were compared using the Mann–Whitney U test; categorical variables were compared using χ^2^ and Fisher’s exact tests. Survival was estimated by the Kaplan–Meier method. Competing risk analysis was used for estimating relapse and NRM, and the Gray’s test was applied for comparisons between subgroups. NRM was the competing event for relapse and *vice versa*. *P* values were two-sided, and *P* < 0.05 was considered to be statistically significant. SPSS 26 (SPSS Inc./IBM, Armonk, NY, USA) and the R software package (version 4.0.0; http://www.r-project.org) were used for data analysis.

## Results

### Patient Characteristics

Among 327 patients achieving ORR after basiliximab treatment, 265 patients achieved DFS at 1 year after HSCT. A total of 231 SR-aGVHD patients were included in the analysis for IR because they offered at least once IR data at 3, 6, 9, or 12 months after HSCT, and their characteristics were shown in **Table 1**. Among them, 188 and 43 patients received <5 doses and ≥5 doses of basiliximab treatments, respectively. The median time from allo-HSCT to basiliximab treatment was 24 (range, 12–95) days, the median interval from the occurrence of aGVHD to basiliximab treatment was 5 (range, 3–25) days, and the median doses of basiliximab were 3 (range, 2–11) doses. A total of 179, 124, 80, and 92 patients were included in the analysis for IR at 3, 6, 9, and 12 months, respectively (**Figure 1**), and their characteristics were compared in **Tables S1–S4**.

**Table 1:** Clinical characteristics of patients included for IR analysis after HID HSCT (n = 231).

Characteristics	Basiliximab any dose (n = 231)	Basiliximab <5 doses (n = 188)	Basiliximab ≥5 doses (n = 43)	*P* value
Age at HID HSCT, n (%)				0.548
<18 years	98 (42.4)	78 (41.5)	20 (46.5)	
≥18 years	133 (57.6)	110 (58.5)	23 (53.5)	
Sex, n (%)				0.985
Male	134 (58.0)	109 (58.0)	25 (58.1)	
Female	97 (42.0)	79 (42.0)	18 (41.9)	
Underlying disease, n (%)				0.424
Acute leukemia	164 (71.0)	134 (71.3)	30 (69.8)	
Myelodysplastic syndrome	13 (5.6)	12 (6.4)	1 (2.3)	
Chronic myeloid leukemia	2 (0.9)	2 (1.1)	0 (0.0)	
Severe aplastic anemia	39 (16.9)	31 (16.5)	8 (18.6)	
Non-Hodgkin’s lymphoma	5 (2.2)	4 (2.1)	1 (2.3)	
Multiple myeloma	2 (0.9)	2 (1.1)	0 (0.0)	
Myeloproliferative neoplasms	3 (1.3)	2 (1.1)	1 (2.3)	
Others	3 (1.3)	1 (0.5)	2 (4.7)	
HCT-CI score, n (%)				0.141
Low risk	193 (83.5)	154 (81.9)	39 (90.7)	
Intermediate risk	28 (12.1)	24 (12.8)	4 (9.3)	
High risk	10 (4.3)	10 (5.3)	0 (0.0)	
Donor–recipient relationship, n (%)				0.655
Father–child	142 (61.5)	116 (61.7)	26 (60.5)	
Mother–child	12 (5.2)	8 (4.3)	4 (9.3)	
Sibling–sibling	38 (16.5)	32 (17.0)	6 (14.0)	
Child–parent	36 (15.6)	30 (16.0)	6 (14.0)	
Collateral related donor	3 (1.3)	2 (1.1)	1 (2.3)	
Donor–recipient sex matched, n (%)				0.832
Male to male	110 (47.6)	91 (48.4)	19 (44.2)	
Male to female	78 (33.8)	64 (34.0)	14 (32.6)	
Female to male	24 (10.4)	18 (9.6)	6 (14.0)	
Female to female	19 (8.2)	15 (8.0)	4 (9.3)	
Graft type, n (%)				0.564
Peripheral blood	4 (1.7)	3 (1.6)	1 (2.3)	
Bone marrow and peripheral blood	227 (98.3)	185 (98.4)	42 (97.7)	
Median mononuclear cell counts, 10^8^/kg (range)	8.6 (4.2–14.8)	8.6 (4.2–14.5)	8.7 (4.7–14.8)	0.450
Median CD34^+^ cell counts, ×10^6^/kg (range)	2.3 (0.1–9.8)	2.3 (0.1–9.8)	2.2 (0.9–6.6)	0.730
Neutrophil engraftment, n (%)	231 (100.0)	188 (100.0)	43 (100.0)	1.000
Median time from HSCT to neutrophil engraftment, days (range)	13 (7–42)	13 (7–42)	12 (9–23)	0.282
Platelet engraftment, n (%)	223 (96.5)	183 (97.3)	40 (93.0)	0.170
Median time from HSCT to platelet engraftment, days (range)	17 (6–267)	18 (6–220)	15 (9–267)	0.611
Severity of aGVHD, at the beginning of the basiliximab treatment, n (%)				0.058
<Grade III	194 (84.0)	162 (86.2)	32 (74.4)	
≥Grade III	37 (16.0)	26 (13.8)	11 (25.6)	
Median follow-up after HSCT, days (range) Median follow-up after basiliximab treatment, days (range)	1,367 (121–2,080) 1,336 (106–2,065)	1,387 (155–2,080) 1,352 (118–2,065)	1,284 (121–1,984) 1,267 (106–1,906)	0.174 0.201

IR, immune reconstitution; HID HSCT, haploidentical donor hematopoietic stem cell transplantation; HCT-CI, Hematopoietic Cell Transplantation–Specific Comorbidity Index; aGVHD, acute graft-versus-host disease.

### Immune Reconstitution at 1 and 2 Months After Haploidentical Donor Hematopoietic Stem Cell Transplantation

**Figure 2:**
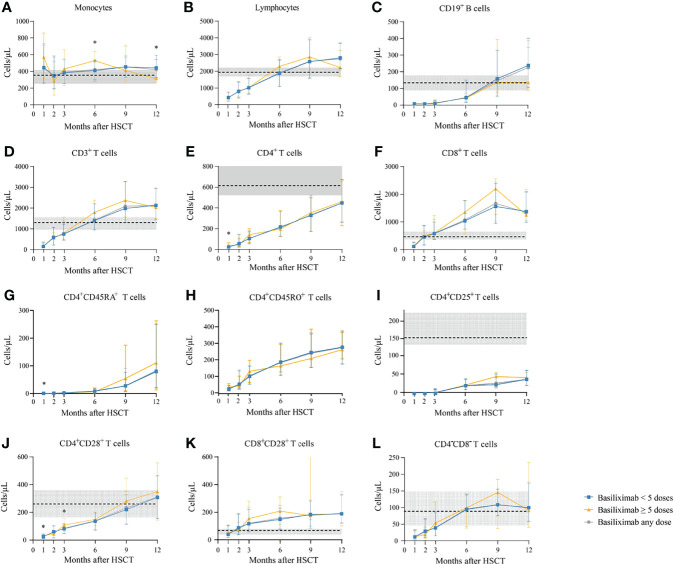
**(A-L)** Kinetics of immune reconstitution in the total population that showed an ORR after basiliximab treatment. Immune cell subsets are compared between basiliximab <5 doses and basiliximab ≥5 doses. Because of lack of data about CD4^+^CD45RA^+^ T cells and CD4^+^CD45RO^+^ T cells in healthy donors, the values of these two immune cell subsets in healthy donors are not shown. Data are shown as median absolute counts with error bars indicating the 25th–75th percentiles. The horizontal dotted lines represent the median value of healthy cohorts, and the gray areas represent the 25th–75th percentiles for the healthy cohorts. *P < 0.05, basiliximab <5 doses vs. basiliximab ≥5 doses. ORR, overall response rate.

**Figure 3:**
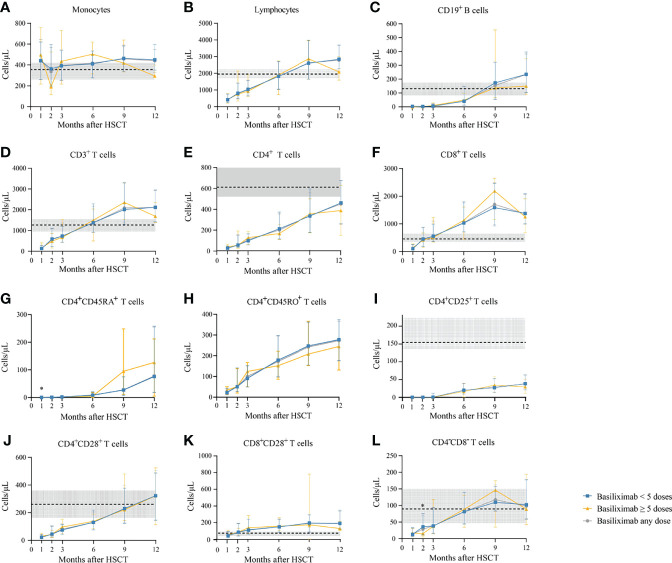
**(A-L)** Kinetics of immune reconstitution in grade II SR-aGVHD patients who showed an ORR after basiliximab treatment. Because of lack of data about CD4^+^CD45RA^+^ T cells and CD4^+^CD45RO^+^ T cells in healthy donors, the values of these two immune cell subsets in healthy donors are not shown. Data are shown as median absolute counts with error bars indicating the 25th–75th percentiles. The horizontal dotted lines represent the median value of healthy cohorts, and the gray areas represent the 25th–75th percentiles for the healthy cohorts. *P < 0.05, basiliximab <5 doses vs. basiliximab ≥5 doses. ORR, overall response rate; SR-aGVHD, steroid-refractory acute graft-versus-host disease.

**Figure 4:**
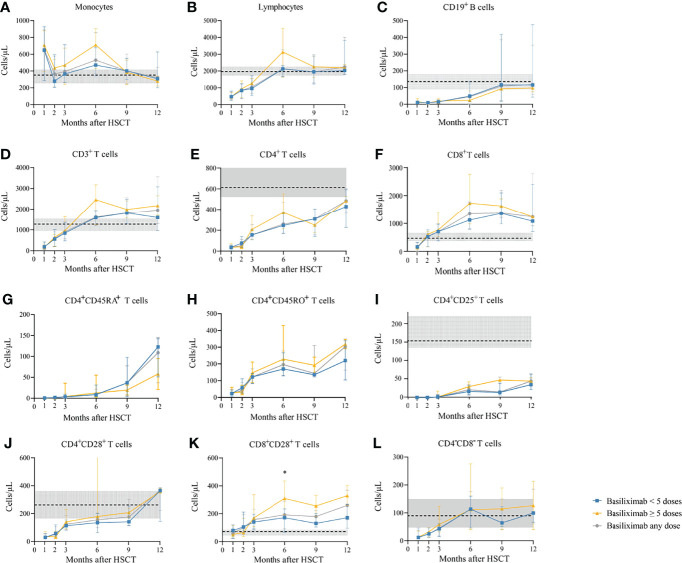
**(A-L)** Kinetics of immune reconstitution in grade III–IV SR-aGVHD patients who showed an ORR after basiliximab treatment. Because of lack of data about CD4^+^CD45RA^+^ T cells and CD4^+^CD45RO^+^ T cells in healthy donors, the values of these two immune cell subsets in healthy donors are not shown. Data are shown as median absolute counts with error bars indicating the 25th–75th percentiles. The horizontal dotted lines represent the median value of healthy cohorts, and the gray areas represent the 25th–75th percentiles for the healthy cohorts. *P < 0.05, basiliximab <5 doses vs. basiliximab ≥5 doses. ORR, overall response rate; SR-aGVHD, steroid-refractory acute graft-versus-host disease.

Among a total of 231 SR-aGVHD patients included in the analysis for IR, 180 and 151 patients had IR data available at 1 and 2 months following HSCT, respectively. The CD4^+^ T cells (*P* = 0.035), CD4^+^naïve T cells (CD4^+^CD45RA^+^ T cells) (*P* = 0.017), and CD4^+^CD28^+^ T cells (*P* = 0.042) were higher in patients receiving ≥5 doses of basiliximab compared with those receiving <5 doses of basiliximab at 1 month after HID HSCT (**Table 2**; **Figure 2**). But they were comparable between patients receiving <5 doses and ≥5 doses of basiliximab at 2 months after HID HSCT (**Table 2**; **Figure 2**). The monocytes, lymphocytes, CD19^+^ B cells, CD3^+^ T cells, CD8^+^ T cells, CD4^+^ memory T cells (CD4^+^CD45RO^+^ T cells), CD4^+^CD25^+^ T cells and CD8^+^CD28^+^ T cells were all comparable between patients receiving <5 doses and ≥5 doses of basiliximab at 1 and 2 months after HID HSCT, which were also observed in both grade II and grade III–IV aGVHD groups (**Table 2**; **Figures 2**–**4**).

**Table 2:** The values of immune cell subset counts at 1 and 2 months after HID HSCT [median (25th–75th)].

	Basiliximab any dose	Basiliximab <5 doses	Basiliximab ≥5 doses	*P* value
1 month	(n = 180^†^)	(n = 146)	(n = 34)	
Monocyte	448.02 (259.47–729.59)	439.46 (255.88–712.39)	567.77 (313.10–856.15)	0.196
Lymphocyte	423.93 (230.29–749.33)	419.96 (230.30–750.81)	429.06 (222.67–750.86)	0.726
CD19^+^ B cell	5.62 (1.97–11.77)	5.92 (2.13–12.23)	5.05 (1.72–10.02)	0.519
CD3^+^ T cell	160.42 (79.31–332.29)	148.68 (77.82–323.55)	172.59 (105.58–389.10)	0.348
CD4^+^ T cell	26.20 (12.88–46.32)	24.07 (12.09–45.75)	37.76 (20.19–66.12)	0.035*
CD8^+^ T cell	106.56 (52.89–257.99)	103.52 (51.44–251.33)	120.44 (63.67–274.21)	0.394
CD4^+^ naïve T cell	0.43 (0.15–0.92)	0.33 (0.13–0.84)	0.60 (0.19–1.93)	0.017*
CD4^+^ memory T cell	24.95 (12.25–45.66)	23.51 (11.22–44.80)	34.44 (17.91–55.96)	0.066
CD4^+^CD25^+^ T cell	0.00 (0.00–1.36)	0.00 (0.00–1.40)	0.00 (0.00–0.46)	0.190
CD4^+^CD28^+^ T cell	23.74 (11.52–45.03)	21.68 (10.22–43.30)	29.38 (16.34–51.13)	0.042*
CD8^+^CD28^+^ T cell	42.89 (19.93–108.03)	42.28 (19.84–109.55)	52.80 (21.39–102.72)	0.388
CD4^–^CD8^–^ T cell	12.17 (5.17–31.56)	11.91 (5.00–33.27)	12.48 (7.26–29.67)	0.602
IgA (G/L)	0.49 (0.37–0.65)	0.49 (0.38–0.64)	0.49 (0.26–0.73)	0.406
IgG (G/L)	11.5 (8.5–16.4)	12.0 (8.6–17.0)	9.6 (7.6–13.3)	0.031^*^
IgM (G/L)	0.390 (0.276–0.555)	0.396 (0.285–0.555)	0.341 (0.241–0.571)	0.189
2 months	(n = 151)	(n = 124)	(n = 27)	
Monocyte	339.60 (183.30–567.00)	344.50 (190.07–583.25)	282.88 (112.32–518.32)	0.242
Lymphocyte	790.92 (432.00–1,351.27)	791.70 (447.93–1,338.19)	790.92 (349.65–1,390.80)	0.977
CD19^+^ B cell	5.46 (2.31–9.90)	5.57 (2.48–10.21)	4.68 (1.35–8.18)	0.452
CD3^+^ T cell	585.50 (231.70–1,054.35)	593.11 (232.75–1,078.25)	551.62 (188.16–819.57)	0.583
CD4^+^ T cell	53.24 (29.56–147.36)	57.75 (32.59–148.55)	44.44 (21.63–103.80)	0.334
CD8^+^ T cell	449.68 (167.83–841.23)	448.87 (168.40–865.63)	490.88 (145.08–669.00)	0.730
CD4^+^ naïve T cell	0.94 (0.34–3.40)	1.00 (0.36–4.11)	0.83 (0.23–1.79)	0.208
CD4^+^ memory T cell	51.02 (27.02–131.00)	52.31 (28.45–138.47)	42.53 (20.20–100.86)	0.395
CD4^+^CD25^+^ T cell	0.00 (0.00–0.33)	0.00 (0.00–0.39)	0.00 (0.00–0.04)	0.109
CD4^+^CD28^+^ T cell	45.54 (21.12–100.80)	46.54 (25.22–109.36)	40.80 (18.72–72.54)	0.346
CD8^+^CD28^+^ T cell	84.79 (38.85–187.60)	90.33 (38.26–191.12)	70.72 (49.92–130.48)	0.683
CD4^–^CD8^–^ T cell	27.30 (10.17–64.23)	29.40 (10.94–67.93)	18.77 (5.85–41.42)	0.088
IgA (G/L)	0.32 (0.17–0.51)	0.33 (0.17–0.51)	0.30 (0.20–0.62)	0.674
IgG (G/L)	11.3 (6.6–15.6)	11.5 (7.2–16.1)	9.5 (5.8–14.5)	0.206
IgM (G/L)	0.290 (0.206–0.453)	0.303 (0.227–0.464)	0.255 (0.178–0.442)	0.260

HID HSCT, haploidentical donor hematopoietic stem cell transplantation; IR, immune reconstitution; SR-aGVHD, steroid-refractory acute graft-versus-host disease.

^*^P < 0.05.

^†^Among a total of 231 SR-aGVHD patients included in the analysis for IR, 180 and 151 patients had IR data available at 1 and 2 months following HSCT, respectively.

**Figure 5:**
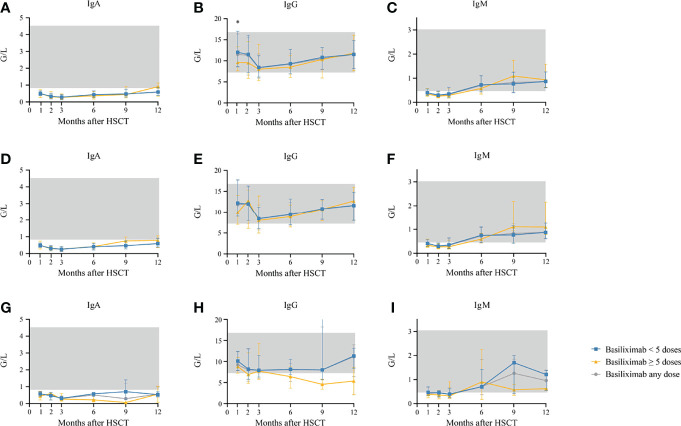
**(A–C)** Kinetics of IgA, IgG, and IgM in the total population who showed an ORR after basiliximab treatment; **(D–F)** Kinetics of IgA, IgG, and IgM in grade II SR-aGVHD patients who showed an ORR after basiliximab treatment; **(G–I)** Kinetics of IgA, IgG, and IgM in grade III–IV SR-aGVHD patients who showed an ORR after basiliximab treatment. IgA, IgG, and IgM are compared between basiliximab <5 doses and basiliximab ≥5 doses. Data are shown as median absolute counts with error bars indicating the 25th–75th percentiles. The gray areas represent the normal range. *P < 0.05, basiliximab <5 doses vs. basiliximab ≥5 doses. ORR, overall response rate; SR-aGVHD, steroid-refractory acute graft-versus-host disease.

Serum IgG was lower in patients receiving ≥5 doses of basiliximab compared with those receiving <5 doses of basiliximab at 1 month after HID HSCT (*P* = 0.031) (**Table 2**; **Figure 5**). However, IgG at 2 months after HID HSCT was comparable between patients receiving <5 doses and ≥5 doses of basiliximab (**Table 2**, **Figure 5B**), which was also observed in both grade II and grade III–IV aGVHD groups (**Figures 5E, H**). Serum IgA and IgM at 1 and 2 months after HID HSCT were all comparable between patients receiving <5 doses and ≥5 doses of basiliximab (**Table 2**, **Figures 5A, C**), which were also observed in both grade II and grade III–IV aGVHD groups (**Figures 5D, F, G, I**).

### Kinetics of Immune Reconstitution After Basiliximab Therapy

The absolute counts of the immune cells at 3, 6, 9, and 12 months after HID HSCT were shown in **Table 3** and **Figures 2**–**4**.

**Table 3:** The values of immune cell subset counts at 3, 6, 9, and 12 months after HID HSCT [median (25th–75th)].

	Basiliximab any dose	Basiliximab <5 doses	Basiliximab ≥5 doses	*P* value
3 months	(n = 179)	(n = 142)	(n = 37)	
Monocyte	387.80 (250.38–549.72)	380.90 (251.54–535.93)	427.80 (228.99–657.94)	0.360
Lymphocyte	1,015.00 (639.73–1,581.72)	1,008.35 (625.13–1,555.76)	1,021.50 (686.23–1,755.75)	0.814
CD19^+^ B cell	8.79 (2.82–25.42)	8.18 (2.73–23.43)	12.04 (3.48–30.99)	0.218
CD3^+^ T cell	761.51 (464.94–1,376.28)	747.32 (446.71–1,226.61)	794.40 (498.51–1,546.74)	0.273
CD4^+^ T cell	108.29 (56.43–179.39)	106.27 (55.18–171.82)	143.40 (68.04– 200.51)	0.110
CD8^+^ T cell	577.98 (364.98–1,085.00)	569.74 (359.64–985.17)	585.15 (394.43–1,230.58)	0.340
CD4^+^ naïve T cell	2.26 (0.91–4.65)	2.23 (0.92–4.45)	2.27 (0.88–6.71)	0.594
CD4^+^ memory T cell	103.37 (53.20–163.53)	99.63 (52.68–162.43)	130.18 (62.61–197.51)	0.138
CD4^+^CD25^+^ T cell	0.61 (0.00–9.69)	0.71 (0.00–11.00)	0.00 (0.00–3.23)	0.068
CD4^+^CD28^+^ T cell	84.78 (48.65–123.55)	78.89 (47.07–118.43)	105.41 (63.14–158.33)	0.046*
CD8^+^CD28^+^ T cell	121.92 (60.33–240.30)	118.75 (59.38–223.18)	156.21 (78.48–283.17)	0.177
CD4^–^CD8^–^ T cell	39.84 (14.82–89.20)	38.92 (14.82–84.36)	53.84 (14.90–116.68)	0.490
IgA (G/L)	0.27 (0.14–0.43)	0.28 (0.13–0.43)	0.27 (0.16–0.47)	0.827
IgG (G/L)	8.3 (5.8–11.6)	8.4 (6.1–11.2)	8.0 (5.3–13.9)	0.897
IgM (G/L)	0.321 (0.188–0.619)	0.348 (0.191–0.627)	0.286 (0.176–0.562)	0.200
6 months	(n = 124)	(n = 104)	(n = 20)	
Monocyte	419.34 (300.19–537.86)	406.97 (282.31–530.24)	527.10 (352.26–637.77)	0.030*
Lymphocyte	1,908.82 (1,079.38–2,699.60)	1,873.53 (1,079.38–2,657.57)	2,289.66 (1,086.41–2,840.60)	0.545
CD19^+^ B cell	42.50 (13.62–137.09)	43.28 (12.92–148.17)	39.66 (18.23–69.14)	0.791
CD3^+^ T cell	1,414.51 (944.52–2,198.74)	1,401.26 (944.52–2,198.74)	1,791.96 (937.98–2,380.90)	0.644
CD4^+^ T cell	211.51 (130.03–366.80)	218.57 (123.38–366.80)	197.67 (130.03–375.17)	0.775
CD8^+^ T cell	1,064.76 (731.89–1,768.82)	1,028.17 (732.35–1,768.82)	1,342.00 (551.82–1,753.35)	0.812
CD4^+^ naïve T cell	8.32 (3.03–19.21)	8.53 (2.97–19.81)	6.30 (3.10–18.36)	0.519
CD4^+^ memory T cell	183.04 (108.88–301.26)	186.18 (104.40–301.26)	163.39 (125.93–292.84)	0.887
CD4^+^CD25^+^ T cell	19.94 (8.67–38.06)	19.64 (9.02–39.14)	21.62 (4.74–36.61)	0.596
CD4^+^CD28^+^ T cell	133.13 (78.90–204.76)	130.64 (73.76–204.89)	151.04 (102.47–192.78)	0.463
CD8^+^CD28^+^ T cell	159.17 (82.55–252.01)	149.96 (75.46–232.78)	209.78 (113.50–312.82)	0.073
CD4^–^CD8^–^ T cell	96.16 (42.67–139.74)	94.52 (44.91–140.48)	99.30 (38.58–136.79)	0.983
IgA (G/L)	0.43 (0.24–0.63)	0.43 (0.24–0.65)	0.36 (0.17–0.62)	0.862
IgG (G/L)	9.2 (6.7–11.8)	9.3 (7.0–12.7)	8.5 (6.0–11.2)	0.155
IgM (G/L)	0.703 (0.430–1.103)	0.724 (0.433–1.103)	0.576 (0.343–1.105)	0.357
9 months	(n = 80)	(n = 71)	(n = 9)	
Monocyte	450.12 (326.55–590.42)	451.08 (329.70–567.92)	411.18 (282.10–702.80)	0.994
Lymphocyte	2,589.32 (1,606.93–3,919.07)	2,569.38 (1,560.21–3,865.29)	2,850.54 (1,614.26–4,023.20)	0.778
CD19^+^ B cell	144.86 (57.08–326.72)	156.66 (49.78–327.75)	134.32 (75.80–393.54)	0.837
CD3^+^ T cell	2,088.98 (1,431.81–3,275.17)	1,982.04 (1,313.76–3,287.99)	2,369.60 (1,566.92–3,261.14)	0.433
CD4^+^ T cell	333.90 (174.57–501.64)	329.60 (174.30–522.89)	354.73 (173.99–498.29)	0.897
CD8^+^ T cell	1,660.99 (997.48–2,432.81)	1,551.48 (932.67–2,382.12)	2,189.42 (1,262.43–2,547.83)	0.244
CD4^+^ naïve T cell	28.03 (7.13–90.50)	27.72 (7.08–76.66)	54.73 (9.52–174.62)	0.470
CD4^+^ memory T cell	239.89 (152.84–364.32)	244.36 (152.72–357.06)	208.15 (153.71–385.56)	0.945
CD4^+^CD25^+^ T cell	26.88 (13.67–54.23)	22.70 (13.19–51.21)	45.26 (16.76–57.21)	0.407
CD4^+^CD28^+^ T cell	212.34 (123.55–361.48)	203.90 (121.38–334.08)	220.79 (156.28–467.87)	0.373
CD8^+^CD28^+^ T cell	181.86 (102.50–289.73)	185.45 (85.44–281.55)	177.37 (149.72–632.63)	0.238
CD4^–^CD8^–^ T cell	108.94 (74.88–157.93)	108.36 (76.25–154.78)	145.44 (36.31–184.45)	0.535
IgA (G/L)	0.47 (0.28–0.75)	0.47 (0.28–0.75)	0.43 (0.23–0.90)	0.843
IgG (G/L)	10.5 (7.9–13.1)	10.8 (8.0–13.1)	10.3 (5.9– 12.5)	0.428
IgM (G/L)	0.824 (0.423–1.260)	0.766 (0.400–1.250)	1.090 (0.707–1.745)	0.161
12 months	(n = 92)	(n = 82)	(n = 10)	
Monocyte	419.70 (315.93–539.18)	438.79 (325.92–590.36)	312.78 (268.92–403.62)	0.020*
Lymphocyte	2,733.00 (2,066.25–3,655.22)	2,797.65 (2,156.88–3,686.85)	2,217.96 (1,665.58–3,244.21)	0.178
CD19^+^ B cell	223.24 (100.09–384.49)	236.32 (105.25–399.89)	134.04 (86.75–345.16)	0.366
CD3^+^ T cell	2,126.90 (1,433.03–2,952.07)	2,126.90 (1,426.01–2,961.63)	2,033.63 (1,508.54–2,957.68)	0.870
CD4^+^ T cell	449.80 (260.36–672.15)	449.08 (264.86–676.25)	462.21 (229.69–660.72)	0.930
CD8^+^ T cell	1,333.38 (990.99–2,063.55)	1,365.80 (971.10–2,081.60)	1,242.40 (1,049.59–2,172.75)	0.990
CD4^+^ naïve T cell	82.70 (19.61–248.85)	79.12 (20.85–251.29)	110.92 (12.55–262.56)	1.000
CD4^+^ memory T cell	274.18 (175.75–373.96)	276.29 (174.29–375.31)	261.38 (205.39–365.77)	0.880
CD4^+^CD25^+^ T cell	37.35 (21.10–61.93)	37.35 (20.87–62.72)	42.39 (19.20–56.61)	0.950
CD4^+^CD28^+^ T cell	336.83 (146.12–451.58)	327.20 (145.16–450.41)	343.09 (146.46–559.93)	0.910
CD8^+^CD28^+^ T cell	190.36 (119.60–333.33)	190.36 (124.90–327.42)	195.08 (102.84–352.15)	0.773
CD4^–^CD8^–^ T cell	98.90 (57.33–177.26)	99.98 (59.24–173.23)	91.26 (41.28–235.88)	0.958
IgA (G/L)	0.59 (0.38–0.94)	0.58 (0.39–0.84)	0.90 (0.33–1.12)	0.547
IgG (G/L)	11.5 (8.1–14.8)	11.5 (8.1–14.8)	11.8 (7.6–16.1)	0.970
IgM (G/L)	0.865 (0.614–1.265)	0.865 (0.600–1.255)	0.935 (0.625–1.578)	0.555

HID HSCT, haploidentical donor hematopoietic stem cell transplantation.

^*^P < 0.05.

#### Monocytes

The monocyte counts achieved a comparable level in healthy controls since the third month after HID HSCT (**Figure 2A**). The monocyte absolute counts were comparable between patients receiving <5 doses and ≥5 doses of basiliximab at 3 and 9 months after HID HSCT (**Figure 2A**); however, it was higher in patients receiving ≥5 doses of basiliximab compared with those receiving <5 doses of basiliximab at 6 months after HID HSCT (*P* = 0.03). In addition, the monocyte absolute counts were lower in patients receiving ≥5 doses of basiliximab compared with those receiving <5 doses of basiliximab at 12 months after HID HSCT (*P* = 0.02). The monocyte absolute counts at all monitoring points were comparable between patients receiving <5 doses and ≥5 doses of basiliximab in both grade II and grade III–IV aGVHD groups (**Figures 3A**, **4A**).

#### Total Lymphocytes

The lymphocyte absolute counts achieved a comparable level in healthy controls since the sixth month after HID HSCT (**Figure 2B**). The lymphocyte absolute counts at 3, 6, 9, and 12 months after HID HSCT were all comparable between patients receiving <5 doses and ≥5 doses of basiliximab (**Figure 2B**), which were also observed in both grade II and grade III–IV aGVHD groups (**Figures 3B**, **4B**).

#### CD19^+^ B Cells

The absolute counts of CD19^+^ B cells achieved comparable levels in healthy controls since the ninth month after HID HSCT (**Figure 2C**). The absolute counts of CD19^+^ B cells at 3, 6, 9, and 12 months after HID HSCT were all comparable between patients receiving <5 doses and ≥5 doses of basiliximab, which were also observed in both grade II and grade III–IV aGVHD groups (**Figures 3C**, **4C**).

#### CD3^+^ T Cells

The absolute counts of CD3^+^ T cells achieved comparable levels in healthy controls since the sixth month after HID HSCT (**Figure 2D**). The absolute counts of CD3^+^ T cells at 3, 6, 9, and 12 months after HID HSCT were all comparable between patients receiving <5 doses and ≥5 doses of basiliximab (**Figure 2D**), which were also observed in both grade II and grade III–IV aGVHD groups (**Figures 3D**, **4D**).

#### CD4^+^ T Cells

The absolute counts of CD4^+^ T cells and CD4^+^CD25^+^ T cells did not achieve comparable levels in healthy controls within 1 year after HID HSCT (**Figures 2E, I**). The absolute counts of CD4^+^CD28^+^ T cells achieved comparable levels in healthy controls since the ninth month after HID HSCT (**Figure 2J**).

The absolute counts of CD4^+^ T cells (**Figure 2E**), CD4^+^CD45RA^+^ T cells (**Figure 2G**), CD4^+^CD45RO^+^ T cells (**Figure 2H**), and CD4^+^CD25^+^ T cells (**Figure 2I**) at all monitoring points and CD4^+^CD28^+^ T cells (**Figure 2J**) at 6, 9, and 12 months after HID HSCT were all comparable between patients receiving <5 doses and ≥5 doses of basiliximab, which were also observed in both grade II and grade III–IV aGVHD groups (**Figures 3E, G–J**, **4E, G–J**). The absolute counts of CD4^+^CD28^+^ T cells of patients receiving ≥5 doses of basiliximab were higher than those receiving <5 doses of basiliximab at 3 months after HID HSCT (*P* = 0.046) (**Figure 2J**), but they were comparable between patients receiving <5 doses and ≥5 doses of basiliximab in either grade II or grade III–IV aGVHD groups (**Figures 3J**, **4J**).

#### CD8^+^ T Cells

The absolute counts of CD8^+^ T cells (**Figure 2F**) and CD8^+^CD28^+^ T cells (**Figure 2K**) both achieved comparable levels in healthy controls since the third month after HID HSCT. The absolute counts of CD8^+^ T cells (**Figure 2F**) and CD8^+^CD28^+^ T cells (**Figure 2K**) at 3, 6, 9, and 12 months after HID HSCT were all comparable between patients receiving <5 doses and ≥5 doses of basiliximab, which were also observed in both grade II and grade III–IV aGVHD groups (**Figures 3F, K**, **4F, K**), except that the absolute count of CD8^+^CD28^+^ T cells at 6 months after HID HSCT was lower in patients receiving <5 doses compared with that of those receiving ≥5 doses of basiliximab in grade III–IV aGVHD groups (*P* = 0.02) (**Figure 4K**).

#### CD4^–^CD8^–^ T Cells

The absolute counts of CD4^–^CD8^–^ T cells achieved comparable levels in healthy controls since the sixth month after HID HSCT (**Figure 2L**). The absolute counts of CD4^–^CD8^–^ T cells at 3, 6, 9, and 12 months after HID HSCT were all comparable between patients receiving <5 doses and ≥5 doses of basiliximab (**Figure 2L**), which were also observed in both grade II and grade III–IV aGVHD groups (**Figures 3L**, **4L**).

#### Serum IgA, IgG, and IgM

Serum IgA (normal range: 0.82–4.53 G/L) did not reach the normal range within 1 year after HID HSCT (**Figure 5A**). Serum IgG (normal range: 7.2–16.8 G/L) and IgM (normal range: 0.460–3.040 G/L) recovered to the normal range since the first and sixth month after HID HSCT, respectively (**Figures 5B, C**). The levels of serum IgA, IgG, and IgM at 3, 6, 9, and 12 months after HID HSCT were all comparable between patients receiving <5 doses and ≥5 doses of basiliximab (**Table 3**; **Figures 5A–C)**, which were also observed in both grade II and grade III–IV aGVHD groups (**Figures 5D–I**).

### Comparison of Immune Reconstitution Between Patients Who Recovered From Steroid-Refractory Acute Graft-Versus-Host Disease After Basiliximab Therapy and Event-Free Haploidentical Donor Hematopoietic Stem Cell Transplantation Recipients

**Figure 6:**
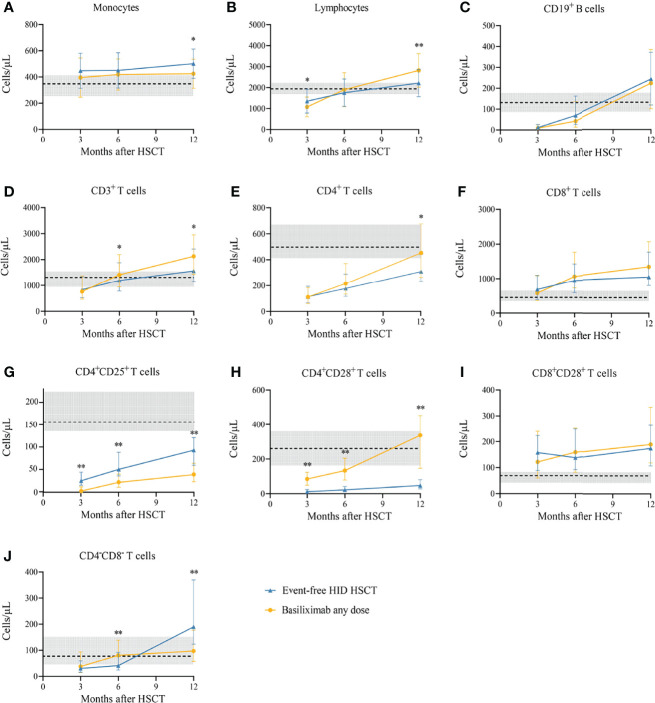
**(A-J)** Immune reconstitution between SR-aGVHD patients who showed an ORR after basiliximab treatment and event-free HID HSCT recipients. Data are shown as median absolute counts with error bars indicating the 25th–75th percentiles. The horizontal dotted lines represent the median value of healthy cohorts, and the gray areas represent the 25th–75th percentiles for the healthy cohorts. *P < 0.05, **P < 0.01, basiliximab any dose vs. event-free HID HSCT. HID HSCT, haploidentical donor hematopoietic stem cell transplantation; ORR, overall response rate; SR-aGVHD, steroid-refractory acute graft-versus-host disease.

The characteristics of IR at 3, 6, and 12 months after HID HSCT between patients who recovered from SR-aGVHD after basiliximab therapy and event-free HID HSCT patients in the historical cohort were shown in **Figure 6**. We observed that the evolution of CD19^+^ B cells, CD8^+^ T cells, and CD8^+^CD28^+^ T cells was all comparable between these two cohorts at each time point. Similarly, the evolution of monocytes, lymphocytes, CD3^+^ T cells, and CD4^+^ T cells was basically comparable between these two cohorts. SR-aGVHD patients who recovered after basiliximab treatment showed a slower recovery of CD4^+^CD25^+^ T cells and a faster recovery of CD4^+^CD28^+^ T cells at each time point compared with those of event-free HID HSCT recipients.

### The Impact of Immune Reconstitution on Prognosis

The infection events of SR-aGVHD patients who recovered after basiliximab treatment were shown in **Tables S5–S10**. At 3 months after HID HSCT, patients with good IR showed a lower rate of any infection, viral infection, and serious bacterial infection than those with poor IR. However, at 6 and 12 months after HID HSCT, the rates of infection were all comparable between patients with and without good IR. The incidences of cGVHD, NRM, DFS, and OS were all comparable between patients with and without good IR (**Tables S11–S13**). In addition, at 3 months after HID HSCT, patients with good IgM IR showed a lower rate of any infection than those with poor IgM IR. However, the infections were all comparable between patients with and without good IR about IgM at 6 and 12 months after HID HSCT, as well as IgA and IgG at any monitoring point after HID HSCT (**Tables S14–S22**).

## Discussion

In the present study, we firstly evaluated the kinetics of IR in patients who recovered from SR-aGVHD after basiliximab treatment following HID HSCT. We observed that 1) IR was fastest for monocytes and CD8^+^ T cells, followed by lymphocytes, CD3^+^ T cells, and CD19^+^ B cells, and slowest for CD4^+^ T cells; 2) Almost all immune cell subsets recovered comparably between patients receiving <5 doses and ≥5 doses of basiliximab; 3) Most immune cell subsets recovered comparably between patients who recovered from SR-aGVHD after basiliximab treatment and event-free patients; 4) All immune cell subsets except CD4^+^ T cells and CD4^+^CD25^+^ T cells achieved comparable levels in healthy donors within 1 year after HID HSCT. To the best of our knowledge, this was the first study to identify the IR in patients who recovered from SR-aGVHD, and our results firstly demonstrated that basiliximab might not have prolonged negative effect on IR in SR-aGVHD survivors.

We observed that in patients who recovered from SR-aGVHD after basiliximab therapy, CD8^+^ T cells rapidly achieved similar levels compared with those of healthy donors and event-free HID HSCT recipients. Basiliximab selectively inhibited activated T lymphocytes, and it did not significantly influence the total peripheral lymphocyte or lymphocyte subset counts (30). Basiliximab did not downregulate IL-2 receptor expression on circulating T lymphocytes either. The terminal elimination half-life of basiliximab was reported to be 13.4 days in adults and 9.4 days in children (31, 32), and the duration of IL-2 receptor saturation with basiliximab was 4–6 weeks and 29 days, respectively, in adults and children (30, 33). Thus, although basiliximab could inhibit the rapid clonal expansion of activated T lymphocytes, it did not show prolonged inhibition on IR of CD8^+^ T cells after the end of basiliximab treatment. Several studies observed that CD8^+^ T cell recovery was associated with improved transplant outcomes. Tian et al. (34) reported that rapid recovery of CD8^+^ T cells was associated with better prognosis. In addition, Pei et al. (21) reported that an extremely high CD8^+^ T cell count (684 cells/µl) was observed in event-free patients at 3 months after HID HSCT. Our results showed that basiliximab did not influence the IR of CD8^+^ T cells in SR-aGVHD survivors, which may contribute to good prognosis of our patients.

In addition, we also observed that monocytes of SR-aGVHD survivors rapidly achieved similar levels compared with those of healthy donors and event-free HID HSCT recipients at most time points. It was because basiliximab did not inhibit the monocyte (35). On the contrary, some second-line therapies, such as ruxolitinib, could significantly inhibit the monocyte (36). Monocyte recovery was important for infection prophylaxis and better survival after allo-HSCT. Podgorny et al. (37) reported that low counts of monocytes (total and inflammatory) were associated with increased viral infections after HSCT. Storek et al. (38) also observed that the counts of the monocytes on day 80 were significantly inversely correlated with the rates of any infection, severe infections, and viral infections between days 100 and 365 after allo-HSCT. In addition, DeCook et al. (39) reported that the absolute count of monocytes in peripheral blood >0.3 × 10^9^/L at day +100 was associated with improved relapse-free survival, and monocyte recovery at day +100 was an independent prognostic factor for improved survival in multivariate analysis. Basiliximab did not inhibit the IR of monocytes, which may partly contribute to the favorable outcomes of SR-aGVHD survivors.

Poorer B cell reconstitution was associated with increased infections after allo-HSCT (38). Corre et al. (40) reported that patients with a slower B-cell recovery at 12 months after HSCT experienced higher rates of late infection. Storek et al. (38) reported that the counts of total B cells on day 80 were significantly inversely correlated with the rates of any infection, viral infections, bacterial infections, and fungal infections between days 100 and 365 after allo-HSCT. Basiliximab treatment did not inhibit the B cells, and we observed that the IR of CD19^+^ B cells in patients who recovered from SR-aGVHD was similar to that in event-free HID HSCT recipients, which may help to prevent the late infections in SR-aGVHD survivors.

Our results showed that serum IgA did not recover to the normal range within 1 year after HID HSCT, while serum IgG and IgM returned to the normal range since the first and sixth month after HID HSCT, respectively. Previous studies showed that IgG and IgM achieved the normal level 3–4 months after HSCT (41–44). Norlin et al. (44) reported that serum IgG reached the normal range since the third month after HSCT in a cohort of 179 recipients. HID HSCT recipients routinely received immunoglobulin (400 mg/kg) on days 1, 11, 21, and 31 after transplantation at our center (27). In addition, serum IgG had a long half-life of approximately 3 weeks (45). The above two points may account for normal IgG levels at 1 and 2 months after HSCT in the present study. Acute GVHD had a negative effect on serum IgG (46). Nevertheless, SR-aGVHD HSCT recipients included in the present study achieved an ORR within 3 months after HSCT and did not experience serious complications, such as serious infections and cGVHD, which may account for normal IgG levels since the third month after HSCT.

We observed that the IR of CD4^+^ T cells, particularly the CD4^+^CD25^+^ T cells, was relatively slow after basiliximab treatment. However, the recovery of CD4^+^ T cells was comparable between patients who recovered from SR-aGVHD after basiliximab treatment and event-free HID HSCT recipients. Pei et al. (21) reported that CD4^+^ T cells recovered slower in HID HSCT recipients compared with those receiving HLA matched donor HSCT, which may be due to the use of ATG for GVHD prophylaxis (47, 48). Therefore, the slower IR of CD4^+^ T cells in our study might not be fully attributed to basiliximab treatment.

CD28-mediated costimulation was very important in T-cell activation; CD4^+^CD28^+^ T cells and CD8^+^CD28^+^ T cells mediated graft-versus-leukemia (GVL) effect to prevent relapse, and CD4^+^CD28^+^ T cells also mediated protection against infections (49, 50). Poor CD4^+^CD25^+^ T cell reconstitution increased the risk of infections (51). We observed that patients who recovered from SR-aGVHD after basiliximab treatment had a slower CD4^+^CD25^+^ T-cell recovery compared with event-free HID HSCT recipients, which was supported by previous studies (52, 53). Chakupurakal et al. (53) observed significant depletion of CD4^+^CD25^+^ T cells in SR-aGVHD patients treated with basiliximab, and Vondran et al. (54) reported that basiliximab downregulated the expression of CD25 on T cells *in vitro*. However, the relatively slow IR of CD4^+^CD25^+^ T cells did not seem to have a negative effect on clinical outcomes after basiliximab treatment in the present study.

Liu et al. (15) reported that SR-aGVHD patients receiving ≥5 doses of basiliximab were more likely to experience infections. Therefore, patients were divided in <5 dose group and ≥5 dose group in the present study.

In this study, the median interval from the occurrence of aGVHD to basiliximab treatment was 5 days. According to the European Society for Blood and Marrow Transplantation (EBMT), the first-line steroid treatment should be continued for 7 days (7). However, some studies showed that 5 days of steroids helped to distinguish patients with a high risk of NRM (55, 56). In addition, the British Committee for Standards in Haematology (BCSH) and the British Society for Bone Marrow Transplantation (BSBMT) also considered no response after 5 days of steroid as SR-aGVHD (57). Therefore, the definition of SR-aGVHD was different, which should be evaluated by prospective studies in the future. Finally, it might be reasonable for patients failing to respond after 5 days of steroids to receive basiliximab treatment because of poor outcomes of SR-aGVHD patients and the relative safety of basiliximab treatment.

There were several limitations in the study. First, we did not routinely detect the function of immune cell subsets. Thus, we could not identify whether basiliximab treatment would affect the function recovery of the immune cells. Second, in this retrospective study, we did not use MFC to monitor the IR of monocytes; however, we could get the absolute count of monocytes from the blood routine examination. Third, we did not monitor the count and function of natural killer (NK) cells regularly in the present study. Fourth, due to lack of CD127 and Forkhead box P3 (FOXP3) in our routine panels for IR monitoring, we were unable to identify the IR of regulatory T cells (Tregs). However, previous studies showed that basiliximab treatment could decrease the counts of Treg, but it did not seem to have a negative impact on the function of Tregs (52–54). Fifth, because the IR monitoring was not performed in real time after basiliximab treatment, we could not explore whether basiliximab induced a transient lymphodepletion and whether IR could be considered as a sign of aGVHD control. Regardless, IR data at 1 and 2 months after HSCT might provide some information for these issues. Sixth, because we wanted to identify the real rules of IR after basiliximab treatment, HSCT recipients with serious complications that may have a negative impact on IR, such as serious infections and cGVHD, were excluded; however, this reflected a relatively selective subgroup of patients, and the IR of the other survivors after basiliximab treatment should be further identified. Finally, the patients’ heterogeneity such as transplant regimen, primary disease, and donor type may have an impact on the results of the study. Further studies enrolling uniform cohorts may help to further identify the IR after basiliximab treatment.

## Conclusion

In conclusion, IR of most of the immune cells was rapid in SR-aGVHD patients who recovered after basiliximab treatment, which was similar to those of event-free HID HSCT recipients. Thus, basiliximab treatment may not seriously impact the IR in SR-aGVHD survivors.

## Data Availability Statement

The raw data supporting the conclusions of this article will be made available by the authors, without undue reservation.

## Ethics Statement

The studies involving human participants were reviewed and approved by The institutional review board of Peking University People’s Hospital. Written informed consent to participate in this study was provided by the participants’ legal guardian/next of kin.

## Author Contributions

X-DM designed the study. X-HZ, L-PX, YW, C-HY, HC, Y-HC, WH, F-RW, J-ZW, X-YP, Y-JC, and K-YL collected the data. X-DM, D-XD and SF analyzed the data and drafted the manuscript. X-DM and X-JH reviewed the manuscript. All authors contributed to the article and approved the submitted version.

## Funding 

This work was supported by the Program of the National Natural Science Foundation of China (grant number 82170208), the Foundation for Innovative Research Groups of the National Natural Science Foundation of China (grant number 81621001), CAMS Innovation Fund for Medical Sciences (CIFMS) (grant number 2019-I2M-5-034), the Key Program of the National Natural Science Foundation of China (grant number 81930004), and the Fundamental Research Funds for the Central Universities.

## Conflict of Interest

The authors declare that the research was conducted in the absence of any commercial or financial relationships that could be construed as a potential conflict of interest.

## Publisher’s Note

All claims expressed in this article are solely those of the authors and do not necessarily represent those of their affiliated organizations, or those of the publisher, the editors and the reviewers. Any product that may be evaluated in this article, or claim that may be made by its manufacturer, is not guaranteed or endorsed by the publisher.

## Abbreviations

allo-HSCT, allogeneic hematopoietic stem cell transplantation; ATG, anti-thymocyte globulin; CR, complete response; CSA, cyclosporine A; DFS, disease-free survival; DLI, donor lymphocyte infusion; GVHD, graft-versus-host disease; HID, haploidentical donor; HLA, human leukocyte antigen; IFI, invasive fungal infection; IL-2, interleukin-2; IR, immune reconstitution; MFC, multiparameter flow cytometry; MMF, mycophenolate mofetil; MTX, methotrexate; NRM, non-relapse mortality; ORR, overall response rate; OS, overall survival; PTLD, posttransplant lymphoproliferative disorder; SR-aGVHD, steroid-refractory acute graft-versus-host disease.

## References

[1] Zhang XH Chen J Han MZ Huang H Jiang EL Jiang M. The Consensus From The Chinese Society of Hematology on Indications, Conditioning Regimens and Donor Selection for Allogeneic Hematopoietic Stem Cell Transplantation: 2021 Update. J Hematol Oncol (2021) 14(1):145. doi: 10.1186/s13045-021-01159-2 34526099 PMC8441240

[2] Xu LP Lu PH Wu DP Sun ZM Liu QF Han MZ. Hematopoietic Stem Cell T Ransplantation Activity in China 2019: A Report From the Chinese Blood and Marrow Transplantation Registry Group. Bone Marrow Transplant (2021) 56(12):2940–2947. doi: 10.1038/s41409-021-01431-6 PMC8385702 34433917

[3] Chang YJ Xu LP Wang Y Zhang XH Chen H Chen YH. Controlled, Randomized, Open-Label Trial of Risk-Stratified Corticosteroid Prevention of Acute Graft-Versus-Host Disease After Haploidentical Transplantation. J Clin Oncol (2016) 34(16):1855–63. doi: 10.1200/JCO.2015.63.8817 27091717

[4] Garnett C Apperley JF Pavlů J. Treatment and Management of Graft-Versus-Host Disease: Improving Response and Survival. Ther Adv Hematol (2013) 4(6):366–78. doi: 10.1177/2040620713489842 PMC3854558 24319572

[5] Martin PJ Rizzo JD Wingard JR Ballen K Curtin PT Cutler C. First- and Second-Line Systemic Treatment of Acute Graft-Versus-Host Disease: Recommendations of the American Society of Blood and Marrow Transplantation. Biol Blood Marrow Transplant (2012) 18(8):1150–63. doi: 10.1016/j.bbmt.2012.04.005 PMC3404151 22510384

[6] Liu WB Sun YQ Zhang XH Xu LP Wang Y Yan CH. [Risk Factors Analysis for Steroid-Resistant Acute Graft Versus Host Disease After Haploidentical Hematopoietic Stem Cell Transplantation]. Zhonghua xue ye xue za zhi = Zhonghua xueyexue zazhi (2020) 41(2):106–11. doi: 10.3760/cma.j.issn.0253-2727.2020.02.004 PMC7357940 32135625

[7] Penack O Marchetti M Ruutu T Aljurf M Bacigalupo A Bonifazi F. Prophylaxis and Management of Graft Versus Host Disease After Stem-Cell Transplantation for Haematological Malignancies: Updated Consensus Recommendations of the European Society for Blood and Marrow Transplantation. Lancet Haematology (2020) 7(2):e157–67. doi: 10.1016/S2352-3026(19)30256-X 32004485

[8] Berger M Biasin E Saglio F Fagioli F. Innovative Approaches to Treat Steroid-Resistant or Steroid Refractory GVHD. Bone Marrow Transplant (2008) 42 Suppl 2:S101–5. doi: 10.1038/bmt.2008.294 18978736

[9] Shen MZ Li JX Zhang XH Xu LP Wang Y Liu KY. Meta-Analysis of Interleukin-2 Receptor Antagonists as the Treatment for Steroid-Refractory Acute Graft-Versus-Host Disease. Front Immunol (2021) 12. doi: 10.3389/fimmu.2021.749266 PMC8490710 34621279

[10] Malard F Huang XJ and Sim JPY. Treatment and Unmet Needs in Steroid-Refractory Acute Graft-Versus-Host Disease. Leukemia (2020) 34(5):1229–40. doi: 10.1038/s41375-020-0804-2 PMC7192843 32242050

[11] Massenkeil G Rackwitz S Genvresse I Rosen O Dörken B Arnold R. Basiliximab is Well Tolerated and Effective in the Treatment of Steroid-Refractory Acute Graft-Versus-Host Disease After Allogeneic Stem Cell Transplantation. Bone Marrow Transplant (2002) 30(12):899–903. doi: 10.1038/sj.bmt.1703737 12476283

[12] Schmidt-Hieber M Fietz T Knauf W Uharek L Hopfenmüller W Thiel E. Efficacy of the Interleukin-2 Receptor Antagonist Basiliximab in Steroid-Refractory Acute Graft-Versus-Host Disease. Br J Haematol (2005) 130(4):568–74. doi: 10.1111/j.1365-2141.2005.05631.x 16098072

[13] Funke VA de Medeiros CR Setúbal DC Ruiz J Bitencourt MA Bonfim CM. Therapy for Severe Refractory Acute Graft-Versus-Host Disease With Basiliximab, a Selective Interleukin-2 Receptor Antagonist. Bone Marrow Transplant (2006) 37(10):961–5. doi: 10.1038/sj.bmt.1705306 16565744

[14] Wang JZ Liu KY Xu LP Liu DH Han W Chen H. Basiliximab for the Treatment of Steroid-Refractory Acute Graft-Versus-Host Disease After Unmanipulated HLA-Mismatched/Haploidentical Hematopoietic Stem Cell Transplantation. Transplant Proc (2011) 43(5):1928–33. doi: 10.1016/j.transproceed.2011.03.044 21693302

[15] Liu SN Zhang XH Xu LP Wang Y Yan CH Chen H. Prognostic Factors and Long-Term Follow-Up of Basiliximab for Steroid-Refractory Acute Graft-Versus-Host Disease: Updated Experience From a Large-Scale Study. Am J Hematol (2020) 95(8):927–36. doi: 10.1016/j.bbmt.2019.10.031 32311156

[16] Tang FF Cheng YF Xu LP Zhang XH Yan CH Han W. Basiliximab as Treatment for Steroid-Refractory Acute Graft-Versus-Host Disease in Pediatric Patients After Haploidentical Hematopoietic Stem Cell Transplantation. Biol Blood Marrow Transplant (2020) 26(2):351–7. doi: 10.1016/j.bbmt.2019.10.031 31704470

[17] [Chinese Consensus of Allogeneic Hematopoietic Stem Cell Transplantation for Hematological Disease (III) -Acute Graft-Versus-Host Disease (2020)]. Zhonghua xue ye xue za zhi = Zhonghua xueyexue zazhi (2020) 41(7):529–36. doi: 10.3760/cma.j.issn.0253-2727.2020.07.001 PMC7449769 32549120

[18] Bondanza A Ruggeri L Noviello M Eikema DJ Bonini C Chabannon C. Beneficial Role of CD8+ T-Cell Reconstitution After HLA-Haploidentical Stem Cell Transplantation for High-Risk Acute Leukaemias: Results From a Clinico-Biological EBMT Registry Study Mostly in the T-Cell-Depleted Setting. Bone Marrow Transplant (2019) 54(6):867–76. doi: 10.1038/s41409-018-0351-x 30531916

[19] Bejanyan N Brunstein CG Cao Q Lazaryan A Luo X Curtsinger J. Delayed Immune Reconstitution After Allogeneic Transplantation Increases the Risks of Mortality and Chronic GVHD. Blood Adv (2018) 2(8):909–22. doi: 10.1182/bloodadvances.2017014464 PMC5916001 29678809

[20] Elmariah H Brunstein CG Bejanyan N. Immune Reconstitution After Haploidentical Donor and Umbilical Cord Blood Allogeneic Hematopoietic Cell Transplantation. Life (Basel, Switzerland) (2021) 11(2):102. doi: 10.3390/life11020102 PMC7911120 33572932

[21] Pei X Zhao X Wang Y Xu L Zhang X Liu K. Comparison of Reference Values for Immune Recovery Between Event-Free Patients Receiving Haploidentical Allografts and Those Receiving Human Leukocyte Antigen-Matched Sibling Donor Allografts. Front Med (2018) 12(2). doi: 10.1007/s11684-017-0548-1 28887808

[22] Chang YJ Zhao XY Huo MR Xu LP Liu DH Liu KY. Immune Reconstitution Following Unmanipulated HLA-Mismatched/Haploidentical Transplantation Compared With HLA-Identical Sibling Transplantation. J Clin Immunol (2012) 32(2):268–80. doi: 10.1007/s10875-011-9630-7 22173879

[23] Shen MZ Zhang XH Xu LP Wang Y Yan CH Chen H. Preemptive Interferon-α Therapy Could Protect Against Relapse and Improve Survival of Acute Myeloid Leukemia Patients After Allogeneic Hematopoietic Stem Cell Transplantation: Long-Term Results of Two Registry Studies. Front Immunol (2022) 13:757002. doi: 10.3389/fimmu.2022.757002 35154096 PMC8831731

[24] Fan S Shen MZ Zhang XH Xu LP Wang Y Yan CH. Preemptive Immunotherapy for Minimal Residual Disease in Patients With T(8;21) Acute Myeloid Leukemia After Allogeneic Hematopoietic Stem Cell Transplantation. Front Oncol (2022) 11:773394. doi: 10.3389/fonc.2021.773394 35070977 PMC8770808

[25] Xu LP Liu KY Liu DH Han W Chen H Chen YH. A Novel Protocol for Haploidentical Hematopoietic SCT Without *In Vitro* T-Cell Depletion in the Treatment of Severe Acquired Aplastic Anemia. Bone marrow Transplant (2012) 47(12):1507–12. doi: 10.1038/bmt.2012.79 22635243

[26] Shen MZ Liu XX Qiu ZY Xu LP Zhang XH Wang Y. Efficacy and Safety of Mesenchymal Stem Cells Treatment for Multidrug-Resistant Graft-Versus-Host Disease After Haploidentical Allogeneic Hematopoietic Stem Cell Transplantation. Ther Adv Hematol (2022) 13:20406207211072838. doi: 10.1177/20406207211072838 35096361 PMC8796067

[27] Huang XJ Liu DH Liu KY Xu LP Chen H Han W. Haploidentical Hematopoietic Stem Cell Transplantation Without *In Vitro* T-Cell Depletion for the Treatment of Hematological Malignancies. Bone Marrow Transplant (2006) 38(4):291–7. doi: 10.1038/sj.bmt.1705445 16883312

[28] Mo XD Hong SD Zhao YL Jiang EL Chen J Xu Y. Basiliximab for Steroid-Refractory Acute Graft-Versus-Host Disease: A Real-World Analysis. Am J Hematol (2022) 97(4):458–69. doi: 10.1002/ajh.26475 35064928

[29] Jagasia MH Greinix HT Arora M Williams KM Wolff D Cowen EW. National Institutes of Health Consensus Development Project on Criteria for Clinical Trials in Chronic Graft-Versus-Host Disease: I. The 2014 Diagnosis and Staging Working Group Report. Biol Blood marrow Transplant (2015) 21(3):389–401. doi: 10.1016/j.bbmt.2014.12.001 25529383 PMC4329079

[30] Onrust SV Wiseman LR. Basiliximab. Drugs (1999) 57(2):207–13. doi: 10.2165/00003495-199957020-00006 10188761

[31] Kovarik JM Rawlings E Sweny P Fernando O Moore R Griffin PJ. Prolonged Immunosuppressive Effect and Minimal Immunogenicity From Chimeric (CD25) Monoclonal Antibody SDZ CHI 621 in Renal Transplantation. Transplant Proc (1996) 28(2):913–4. 8623459

[32] Kovarik J Mentser M Broyer P Loirat C Crocker J Offner G. DISPOSITION OF BASILIXIMAB, A CHIMERIC IL-2 RECEPTOR (CD25) MONOCLONAL ANTIBODY, IN PEDIATRIC RENAL TRANSPLANT PATIENTS. Transplantation (1998) 65(Supplement):142. doi: 10.1097/00007890-199805131-00252 9448161

[33] Amlot PL Rawlings E Fernando ON Griffin PJ Heinrich G Schreier MH. Prolonged Action of a Chimeric Interleukin-2 Receptor (CD25) Monoclonal Antibody Used in Cadaveric Renal Transplantation. Transplantation (1995) 60(7):748–56. doi: 10.1097/00007890-199510150-00023 7570988

[34] Tian DM Wang Y Zhang XH Liu KY Huang XJ Chang YJ. Rapid Recovery of CD3+CD8+ T Cells on Day 90 Predicts Superior Survival After Unmanipulated Haploidentical Blood and Marrow Transplantation. PloS One (2016) 11(6):e0156777. doi: 10.1371/journal.pone.0156777 27276058 PMC4898737

[35] Pascual J Marcén R Ortuño J. Anti-Interleukin-2 Receptor Antibodies: Basiliximab and Daclizumab. Nephrology Dialysis Transplant (2001) 16(9):1756–60. doi: 10.1093/ndt/16.9.1756 11522853

[36] Ryu DB Lim JY Kim TW Shin S Lee SE Park G. Preclinical Evaluation of JAK1/2 Inhibition by Ruxolitinib in a Murine Model of Chronic Graft-Versus-Host Disease. Exp Hematol (2021) 98:36–46. doi: 10.1016/j.exphem.2021.03.004 33811972

[37] Podgorny PJ Pratt LM Liu Y Dharmani-Khan P Luider J Auer-Grzesiak I. Low Counts of B Cells, Natural Killer Cells, Monocytes, Dendritic Cells, Basophils, and Eosinophils are Associated With Postengraftment Infections After Allogeneic Hematopoietic Cell Transplantation. Biol Blood marrow Transplant (2016) 22(1):37–46. doi: 10.1016/j.bbmt.2015.09.003 26363444

[38] Storek J Espino G Dawson MA Storer B Flowers ME Maloney DG. Low B-Cell and Monocyte Counts on Day 80 are Associated With High Infection Rates Between Days 100 and 365 After Allogeneic Marrow Transplantation. Blood (2000) 96(9):3290–3. doi: 10.1182/blood.V96.9.3290 11050018

[39] DeCook LJ Thoma M Huneke T Johnson ND Wiegand RA Patnaik MM. Impact of Lymphocyte and Monocyte Recovery on the Outcomes of Allogeneic Hematopoietic SCT With Fludarabine and Melphalan Conditioning. Bone Marrow Transplant (2013) 48(5):708–14. doi: 10.1038/bmt.2012.211 23103674

[40] Corre E Carmagnat M Busson M de Latour RP Robin M Ribaud P. Long-Term Immune Deficiency After Allogeneic Stem Cell Transplantation: B-Cell Deficiency is Associated With Late Infections. Haematologica (2010) 95(6):1025–9. doi: 10.3324/haematol.2009.018853 PMC2878804 20133894

[41] Halterman RH Graw RG Jr Fuccillo DA Leventhal BG. Immunocompetence Following Allogeneic Bone Marrow Transplantation in Man. Transplantation (1972) 14(6):689–97. doi: 10.1097/00007890-197212000-00004 4404985

[42] Fass L Ochs HD Thomas ED Mickelson E Storb R Fefer A. Studies of Immunological Reactivity Following Syngeneic or Allogeneic Marrow Grafts in Man. Transplantation (1973) 16(6):630–40. doi: 10.1097/00007890-197312000-00015 4356867

[43] Small TN Keever CA Weiner-Fedus S Heller G O'Reilly RJ Flomenberg N. B-Cell Differentiation Following Autologous, Conventional, or T-Cell Depleted Bone Marrow Transplantation: A Recapitulation of Normal B-Cell Ontogeny. Blood (1990) 76(8):1647–56. doi: 10.1182/blood.V76.8.1647.1647 1698484

[44] Norlin AC Sairafi D Mattsson J Ljungman P Ringdén O Remberger M. Allogeneic Stem Cell Transplantation: Low Immunoglobulin Levels Associated With Decreased Survival. Bone Marrow Transplant (2008) 41(3):267–73. doi: 10.1038/sj.bmt.1705892 17994123

[45] Grevys A Frick R Mester S Flem-Karlsen K Nilsen J Foss S. Antibody Variable Sequences Have a Pronounced Effect on Cellular Transport and Plasma Half-Life. iScience (2022) 25(2):103746. doi: 10.1016/j.isci.2022.103746 35118359 PMC8800109

[46] Koh S Koh H Nanno S Okamura H Nakashima Y Nakamae M. Kinetics of IgG Subclasses and Their Effects on the Incidence of Infection After Allogeneic Hematopoietic Stem Cell Transplantation. Transpl Immunol (2021) 67:101413. doi: 10.1016/j.trim.2021.101413 34022326

[47] Nishihori T Al-Kadhimi Z Hamadani M Kharfan-Dabaja MA. Antithymocyte Globulin in Allogeneic Hematopoietic Cell Transplantation: Benefits and Limitations. Immunotherapy (2016) 8(4):435–47. doi: 10.2217/imt.15.128 26973125

[48] Cherkassky L Lanning M Lalli PN Czerr J Siegel H Danziger-Isakov L. Evaluation of Alloreactivity in Kidney Transplant Recipients Treated With Antithymocyte Globulin Versus IL-2 Receptor Blocker. Am J Transplant (2011) 11(7):1388–96. doi: 10.1111/j.1600-6143.2011.03540.x PMC3226763 21564525

[49] Barrett AJ. Mechanisms of the Graft-Versus-Leukemia Reaction. Stem Cells (1997) 15(4):248–58. doi: 10.1002/stem.150248 9253108

[50] Arens R Loewendorf A Her MJ Schneider-Ohrum K Shellam GR Janssen E. B7-Mediated Costimulation of CD4 T Cells Constrains Cytomegalovirus Persistence. J Virol (2011) 85(1):390–6. doi: 10.1128/JVI.01839-10 PMC3014178 20980516

[51] Zhou JR Shi DY Wei R Wang Y Yan CH Zhang XH. Co-Reactivation of Cytomegalovirus and Epstein-Barr Virus Was Associated With Poor Prognosis After Allogeneic Stem Cell Transplantation. Front Immunol (2021) 11. doi: 10.3389/fimmu.2020.620891 PMC7921792 33664733

[52] López-Abente J Martínez-Bonet M Bernaldo-de-Quirós E Camino M Gil N Panadero E. Basiliximab Impairs Regulatory T Cell (TREG) Function and Could Affect the Short-Term Graft Acceptance in Children With Heart Transplantation. Sci Rep (2021) 11(1):827. doi: 10.1038/s41598-020-80567-9 33436905 PMC7803770

[53] Chakupurakal G García-Márquez MA Shimabukuro-Vornhagen A Theurich S Holtick U Hallek M. Immunological Effects in Patients With Steroid-Refractory Graft-Versus-Host Disease Following Treatment With Basiliximab, a CD25 Monoclonal Antibody. Eur J Haematol (2016) 97(2):121–7. doi: 10.1111/ejh.12691 26492560

[54] Vondran FW Timrott K Tross J Kollrich S Schwarz A Lehner F. Impact of Basiliximab on Regulatory T-Cells Early After Kidney Transplantation: Down-Regulation of CD25 by Receptor Modulation. Transplant Int (2010) 23(5):514–23. doi: 10.1111/j.1432-2277.2009.01013.x 19951265

[55] Van Lint MT Uderzo C Locasciulli A Majolino I Scimé R Locatelli F. Early Treatment of Acute Graft-Versus-Host Disease With High- or Low-Dose 6-Methylprednisolone: A Multicenter Randomized Trial From the Italian Group for Bone Marrow Transplantation. Blood (1998) 92:2288–93. 9746766

[56] Van Lint MT Milone G Leotta S Uderzo C Scimè R Dallorso S. Treatment of Acute Graft-Versus-Host Disease With Prednisolone: Significant Survival Advantage for Day +5 Responders and No Advantage for Nonresponders Receiving Anti-Thymocyte Globulin. Blood (2006) 107:4177–81. doi: 10.1182/blood-2005-12-4851 16449522

[57] Dignan FL Clark A Amrolia P Cornish J Jackson G Mahendra P. Diagnosis and Management of Acute Graft-Versus-Host Disease. Br J Haematol (2012) 158(1):30–45. 22533831 10.1111/j.1365-2141.2012.09129.x

