# Supplementary material for: Immune Reconstitution of Patients Who Recovered From Steroid-Refractory Acute Graft-Versus-Host Disease After Basiliximab Treatment
Source: Front Oncol. 2022 Jul 15;12:916442. doi: 10.3389/fonc.2022.916442 (PMC9351448; doi:10.3389/fonc.2022.916442)
Supplement: Supplementary file 1 [file DataSheet_1.docx]

**Supplementary Appendix**

**Contents**

**Supplementary methods**

Treatment of aGVHD……………………………………………….……………………………………2

Protocol of basiliximab treatment ………….…………………………………….………………………3

Immunophenotyping………………………………………………….………………………………….4

Definitions of clinical outcomes….…………………….………………………………………………..5

**Supplementary Tables**

Table S1. Clinical characteristics of patients included for IR analysis at 3 months after HID HSCT (n=179) ………………………………………………………………………………………….……....6

Table S2. Clinical characteristics of patients included for IR analysis at 6 months after HID HSCT (n=124) ………………………………………...………………………………………………..…........9

Table S3. Clinical characteristics of patients included for IR analysis at 9 months after HID HSCT (n=80) ………………………………………………………………………………………...………...12

Table S4. Clinical characteristics of patients included for IR analysis at 12 months after HID HSCT (n=92) ………………………………………………………………………………………...……...…15

Table S5. Infections after 3 months following HID HSCT………………………………………………18

Table S6. Infections after 6 months following HID HSCT………………………………………….….19

Table S7. Infections after 12 months following HID HSCT…………………………….......………….20

Table S8. Infections between patients with good and poor IR at 3 months after HID HSCT…………...21

Table S9. Infections between patients with good and poor IR at 6 months after HID HSCT…….…….22

Table S10. Infections between patients with good and poor IR at 12 months after HID HSCT…………23

Table S11. Clinical outcomes between patients with good and poor IR at 3 months after HID HSCT…24

Table S12. Clinical outcomes between patients with good and poor IR at 6 months after HID HSCT…25

Table S13. Clinical outcomes between patients with good and poor IR at 12 months after HID HSCT…26

Table S14. Infections between patients with good and poor IgA IR at 3 months after HID HSCT………27

Table S15. Infections between patients with good and poor IgG IR at 3 months after HID HSCT………28

Table S16. Infections between patients with good and poor IgM IR at 3 months after HID HSCT……...29

Table S17. Infections between patients with good and poor IgA IR at 6 months after HID HSCT……....30

Table S18. Infections between patients with good and poor IgG IR at 6 months after HID HSCT………31

Table S19. Infections between patients with good and poor IgM IR at 6 months after HID HSCT……...32

Table S20. Infections between patients with good and poor IgA IR at 12 months after HID HSCT…….33

Table S21. Infections between patients with good and poor IgG IR at 12 months after HID HSCT……..34

Table S22. Infections between patients with good and poor IgM IR at 12 months after HID HSCT…….35

Table S23. Immune reconstitution in SR–aGVHD patients who had immune reconstitution data at all 4 monitoring points (median (25th–75th))………………………………………………………………..36

Table S24. Immune reconstitution between SR-aGVHD patients who had immune reconstitution data at all 4 monitoring points and event-free HID HSCT recipients (median (25th–75th))……………………39

Table S25. Immune reconstitution between SR-aGVHD patients and event-free HID HSCT recipients at 1 and 2 months after HSCT (median (25th–75th))……………………………………………………....40

Table S26. The percentage of immune cell subset at 3, 6, 9, and 12 months after HID HSCT (median (25th–75th))…………………………………………………………………………………………….41

**Treatment of aGVHD**

Once aGVHD was diagnosed, cyclosporine A (CsA) was maintained at therapeutically effective concentration, and methylprednisolone (MP) was used as the first-line treatment for aGVHD. MP usage was 2 mg·kg−1·day−1 for patients aged <10 years or weighing < 30 kg and 1 mg·kg−1·day−1 for others. If SR-aGVHD was diagnosed, basiliximab treatment as monotherapy was started on the basis of CsA and MP.

**Protocol of basiliximab treatment**

Basiliximab (Simulect; Novartis Pharma AG, Basel, Switzerland) was administered at a dose of 20 mg·day^−1^ for children weighing 35 kg or more or adults, and at 10 mg·day^−1^ for children weighing less than 35 kg. Basiliximab was administered on days 1 and 3. Afterward, the injection was repeated weekly until aGVHD was less than grade II. In general, institutional guideline recommended tapering by 30% every 5 days for steroid dosage and stopping within 4 weeks. If the patients had achieved PR at 4 weeks, the basiliximab treatment could be continued at weekly until aGVHD was less than grade II. If there was no response after 4 doses of basiliximab, the patients could be switched to another treatments. However, considering there were no other second-line treatments which were definitely superior to basiliximab, prolonged treatment with basiliximab was also permitted at the request of patients.

**Immunophenotyping**

Peripheral blood samples were obtained from patients at 1, 2, 3, 6, 9, and 12 months after haploidentical donor hematopoietic stem cell transplantation (HID HSCT). A panel of antibody combinations were used to recognize CD3, CD4, CD8, CD45RA, CD45RO, CD19, CD25 and CD28. Multiparameter flow cytometry (MFC) was performed by BD FACSSort machine (Becton Dickinson Biosciences, San Jose, CA, USA). And CellQuest software (BD Biosciences) was used for data analysis.

**Definitions of clinical outcomes**

Relapse was defined as the recurrence of >5% bone marrow blasts, the reappearance of blasts in the blood or the development of extramedullary disease. Non-relapse mortality (NRM) was defined as death without relapse or disease progression. Disease-free survival (DFS) was defined as survival period without relapse or death. Overall survival (OS) was defined as survival period without death of any cause.

**Table S1. Clinical characteristics of patients included for IR analysis at 3 months after HID HSCT (n=179)**

| **Characteristics** | Basiliximab any dose  (n=179^#^) | Basiliximab < 5 doses  (n=142) | Basiliximab ≥ 5 doses (n=37) | *P* value |
| --- | --- | --- | --- | --- |
| Age at HID HSCT, n (%) |  |  |  | 0.403 |
| <18 years | 81 (45.3) | 62 (43.7) | 19 (51.4) |  |
| ≥18 years | 98 (54.7) | 80 (56.3) | 18 (48.6) |  |
| Sex, n (%) |  |  |  | 0.563 |
| Male | 109 (60.9) | 88 (62.0) | 21 (56.8) |  |
| Female | 70 (39.1) | 54 (38.0) | 16 (43.2) |  |
| Underlying disease, n (%) |  |  |  | 0.086 |
| Acute leukemia | 133 (74.3) | 108 (76.1) | 25 (67.6) |  |
| Myelodysplastic syndrome | 8 (4.5) | 8 (5.6) | 0 (0.0) |  |
| Chronic myeloid leukemia | 2 (1.1) | 2 (1.4) | 0 (0.0) |  |
| Severe aplastic anemia | 26 (14.5) | 18 (12.7) | 8 (21.6) |  |
| Non-Hodgkin's lymphoma | 4 (2.2) | 3 (2.1) | 1 (2.7) |  |
| Multiple myeloma | 2 (1.1) | 2 (1.4) | 0 (0.0) |  |
| Myeloproliferative neoplasms | 1 (0.6) | 0 (0.0) | 1 (2.7) |  |
| Others | 3 (1.7) | 1 (0.7) | 2 (5.4) |  |
| HCT-CI score, n (%) |  |  |  | 0.242 |
| Low risk | 149 (83.2) | 116 (81.7) | 33 (89.2) |  |
| Intermediate risk | 22 (12.3) | 18 (12.7) | 4 (10.8) |  |
| High risk | 8 (4.5) | 8 (5.6) | 0 (0.0) |  |
| Donor–recipient relationship, n (%) |  |  |  | 0.556 |
| Father–child | 115 (64.2) | 92 (64.8) | 23 (62.2) |  |
| Mother–child | 10 (5.6) | 6 (4.2) | 4 (10.8) |  |
| Sibling–sibling | 30 (16.8) | 25 (17.6) | 5 (13.5) |  |
| Child–parent | 22 (12.3) | 17 (12.0) | 5 (13.5) |  |
| Collateral related donor | 2 (1.1) | 2 (1.4) | 0 (0.0) |  |
| Donor-recipient sex matched, n (%) |  |  |  | 0.559 |
| Male to male | 90 (50.3) | 74 (52.1) | 16 (43.2) |  |
| Male to female | 57 (31.8) | 45 (31.7) | 12 (32.4) |  |
| Female to male | 19 (10.6) | 14 (9.9) | 5 (13.5) |  |
| Female to female | 13 (7.3) | 9 (6.3) | 4 (10.8) |  |
| Graft type, n (%) |  |  |  | 0.503 |
| Peripheral blood | 3 (1.7) | 2 (1.4) | 1 (2.7) |  |
| Bone marrow and peripheral blood | 176 (98.3) | 140 (98.6) | 36 (97.3) |  |
| Median mononuclear cell counts,  ×10^8^/kg (range) | 8.7 (4.2-14.8) | 8.6 (4.2-14.5) | 9.1 (4.7-14.8) | 0.197 |
| Median CD34+ cell counts,  ×10^6^/kg (range) | 2.3 (0.1-9.8) | 2.3 (0.1-9.8) | 2.3 (1.0-6.6) | 0.449 |
| Neutrophil engraftment, n (%) | 179 (100.0) | 142 (100.0) | 37 (100.0) | 1.000 |
| Median time from HSCT to  neutrophil engraftment, d (range) | 13 (7-42) | 13 (7-42) | 12 (9-21) | 0.054 |
| Platelet engraftment, n (%) | 172 (95.5) | 137 (96.4) | 35 (94.5) | 0.599 |
| Median time from HSCT to platelet  engraftment, d (range) | 16 (8-267) | 18 (8-220) | 16 (9-267) | 0.759 |
| Severity of aGVHD, at the  beginning of the basiliximab  treatment, n (%) |  |  |  | 0.109 |
| < Grade Ⅲ | 155 (86.6) | 126 (88.7) | 29 (78.4) |  |
| ≥ Grade Ⅲ | 24 (13.4) | 16 (11.3) | 8 (21.6) |  |
| Median follow-up after HSCT, d  (range) | 1358 (121-2080) | 1367 (155-2080) | 1284 (121-1984) | 0.313 |
| Median follow-up after basiliximab  treatment, d (range) | 1333 (106-2065) | 1336 (118-2065) | 1267 (106-1906) | 0.252 |

IR, immune reconstitution; HID HSCT, haploidentical donor hematopoietic stem cell transplantation; HCT-CI, hematopoietic cell transplantation–specific comorbidity index; aGVHD, acute graft-versus-host disease. ^#^Among a total of 327 patients achieving ORR, 179 were included for IR analysis at 3 months after HSCT, and the other 148 patients were excluded for the following situation: 1) 4 achieved ORR beyond 3 months after HSCT; 2) 6 suffered aGVHD recurrence; 3) 30 had serious infections; 4) 2 had DLI; 5) 1 had serious cGVHD; 6) 5 relapsed; 7) 2 suffered NRM; 8) 98 lacked of IR data.

**Table S2. Clinical characteristics of patients included for IR analysis at 6 months after HID HSCT (n=124)**

| **Characteristics** | Basiliximab any dose  (n=124^#^) | Basiliximab < 5 doses  (n=104) | Basiliximab ≥ 5 doses (n=20) | *P* value |
| --- | --- | --- | --- | --- |
| Age at HID HSCT, n (%) |  |  |  | 0.937 |
| <18 years | 61 (49.2) | 51 (49.0) | 10 (50.0) |  |
| ≥18 years | 63 (50.8) | 53 (51.0) | 10 (50.0) |  |
| Sex, n (%) |  |  |  | 0.833 |
| Male | 77 (62.0) | 65 (62.5) | 12 (60.0) |  |
| Female | 47 (38.0) | 39 (37.5) | 8 (40.0) |  |
| Underlying disease, n (%) |  |  |  | 0.209 |
| Acute leukemia | 83 (66.9) | 71 (68.3) | 12 (60.0) |  |
| Myelodysplastic syndrome | 9 (7.3) | 9 (8.7) | 0 (0.0) |  |
| Chronic myeloid leukemia | 2 (1.6) | 2 (1.9) | 0 (0.0) |  |
| Severe aplastic anemia | 23 (18.5) | 17 (16.3) | 6 (30.0) |  |
| Non-Hodgkin's lymphoma | 3 (2.4) | 2 (1.9) | 1 (5.0) |  |
| Multiple myeloma | 1 (0.8) | 1 (0.9) | 0 (0.0) |  |
| Myeloproliferative neoplasms | 2 (1.6) | 2 (1.9) | 0 (0.0) |  |
| Others | 1 (0.8) | 0 (0.0) | 1 (5.0) |  |
| HCT-CI score, n (%) |  |  |  | 0.029* |
| Low risk | 103 (83.1) | 83 (79.8) | 20 (100.0) |  |
| Intermediate risk | 16 (12.9) | 16 (15.4) | 0 (0.0) |  |
| High risk | 5 (4.0) | 5 (4.8) | 0 (0.0) |  |
| Donor–recipient relationship, n (%) |  |  |  | 0.381 |
| Father–child | 77 (62.1) | 66 (63.5) | 11 (55.0) |  |
| Mother–child | 6 (4.8) | 4 (3.8) | 2 (10.0) |  |
| Sibling–sibling | 20 (16.1) | 17 (16.3) | 3 (15.0) |  |
| Child–parent | 19 (15.3) | 16 (15.4) | 3 (15.0) |  |
| Collateral related donor | 2 (1.6) | 1 (1.0) | 1 (5.0) |  |
| Donor-recipient sex matched, n (%) |  |  |  | 0.616 |
| Male to male | 65 (52.4) | 56 (53.8) | 9 (45.0) |  |
| Male to female | 34 (27.4) | 29 (27.9) | 5 (25.0) |  |
| Female to male | 12 (9.7) | 9 (8.7) | 3 (15.0) |  |
| Female to female | 13 (10.5) | 10 (9.6) | 3 (15.0) |  |
| Graft type, n (%) |  |  |  | 0.413 |
| Peripheral blood | 3 (2.4) | 2 (2.0) | 1 (5.0) |  |
| Bone marrow and peripheral blood | 121 (97.6) | 102 (98.0) | 19 (95.0) |  |
| Median mononuclear cell counts,  ×10^8^/kg (range) | 8.6 (4.2-14.8) | 8.6 (4.2-14.5) | 8.2 (4.7-14.8) | 0.970 |
| Median CD34+ cell counts,  ×10^6^/kg (range) | 2.3 (0.1-5.9) | 2.3 (0.1-5.9) | 2.1 (0.9-5.3) | 0.480 |
| Neutrophil engraftment, n (%) | 124 (100.0) | 104 (100.0) | 20 (100.0) | 1.000 |
| Median time from HSCT to  neutrophil engraftment, d (range) | 13 (8-42) | 13 (8-42) | 12 (10-23) | 0.532 |
| Platelet engraftment, n (%) | 121 (97.5) | 102 (98.0) | 19 (95.0) | 0.414 |
| Median time from HSCT to platelet  engraftment, d (range) | 18 (6-220) | 19 (6-220) | 16 (9-198) | 0.930 |
| Severity of aGVHD, at the  beginning of the basiliximab  treatment, n (%) |  |  |  | 0.093 |
| < Grade Ⅲ | 104 (83.9) | 90 (86.5) | 14 (70.0) |  |
| ≥ Grade Ⅲ | 20 (16.1) | 14 (13.5) | 6 (30.0) |  |
| Median follow-up after HSCT, d  (range) | 1350.5 (198-2054) | 1367 (267-2054) | 1257 (198-1984) | 0.097 |
| Median follow-up after basiliximab  treatment, d (range) | 1304 (114-2035) | 1342 (244-2035) | 1229 (114-1906) | 0.121 |

IR, immune reconstitution; HID HSCT, haploidentical donor hematopoietic stem cell transplantation; HCT-CI, hematopoietic cell transplantation–specific comorbidity index; aGVHD, acute graft-versus-host disease. * means *P* < 0.05.^#^Among a total of 327 patients achieving ORR, 124 were included for IR analysis at 6 months after HSCT, and the other 203 patients were excluded for the following situation: 1) 6 suffered aGVHD recurrence; 2) 52 had serious infections; 3) 5 had DLI; 4) 1 received second HSCT; 5) 51 had serious cGVHD; 6) 14 relapsed; 7) 3 suffered NRM; 8) 71 lacked of IR data.

**Table S3. Clinical characteristics of patients included for IR analysis at 9 months after HID HSCT (n=80)**

| **Characteristics** | Basiliximab any dose  (n=80^#^) | Basiliximab < 5 doses (n=71) | Basiliximab ≥ 5 doses  (n=9) | *P* value |
| --- | --- | --- | --- | --- |
| Age at HID HSCT, n (%) |  |  |  | 1.000 |
| <18 years | 41 (43.8) | 36 (50.7) | 5 (55.6) |  |
| ≥18 years | 39 (56.2) | 35 (49.3) | 4 (44.4) |  |
| Sex, n (%) |  |  |  | 0.630 |
| Male | 52 (65.0) | 45 (63.4) | 7 (77.8) |  |
| Female | 28 (35.0) | 26 (36.6) | 2 (22.2) |  |
| Underlying disease, n (%) |  |  |  | 0.881 |
| Acute leukemia | 64 (80.0) | 56 (78.9) | 8 (88.9) |  |
| Myelodysplastic syndrome | 6 (7.5) | 6 (8.5) | 0 (0.0) |  |
| Chronic myeloid leukemia | 1 (1.3) | 1 (1.4) | 0 (0.0) |  |
| Severe aplastic anemia | 6 (7.5) | 5 (7.0) | 1 (11.1) |  |
| Non-Hodgkin's lymphoma | 1 (1.3) | 1 (1.4) | 0 (0.0) |  |
| Multiple myeloma | 0 (0.0) | 0 (0.0) | 0 (0.0) |  |
| Myeloproliferative neoplasms | 2 (2.5) | 2 (2.8) | 0 (0.0) |  |
| Others | 0 (0.0) | 0 (0.0) | 0 (0.0) |  |
| HCT-CI score, n (%) |  |  |  | 0.779 |
| Low risk | 69 (86.3) | 61 (85.9) | 8 (88.9) |  |
| Intermediate risk | 8 (10.0) | 7 (9.9) | 1 (11.1) |  |
| High risk | 3 (3.7) | 3 (4.2) | 0 (0.0) |  |
| Donor–recipient relationship, n (%) |  |  |  | 0.911 |
| Father–child | 52 (65.0) | 46 (64.8) | 6 (66.7) |  |
| Mother–child | 1 (1.3) | 1 (1.4) | 0 (0.0) |  |
| Sibling–sibling | 14 (17.5) | 12 (16.9) | 2 (22.2) |  |
| Child–parent | 11 (13.7) | 10 (14.1) | 1 (11.1) |  |
| Collateral related donor | 2 (2.5) | 2 (2.8) | 0 (0.0) |  |
| Donor-recipient sex matched, n (%) |  |  |  | 0.596 |
| Male to male | 45 (56.2) | 38 (53.5) | 7 (77.8) |  |
| Male to female | 20 (25.0) | 19 (26.8) | 1 (11.1) |  |
| Female to male | 7 (8.8) | 7 (9.8) | 0 (0.0) |  |
| Female to female | 8 (10.0) | 7 (9.8) | 1 (11.1) |  |
| Graft type, n (%) |  |  |  | 0.112 |
| Peripheral blood | 1 (1.3) | 0 (0.0) | 1 (11.1) |  |
| Bone marrow and peripheral blood | 79 (98.7) | 71 (100.0) | 8 (88.9) |  |
| Median mononuclear cell counts,  ×10^8^/kg (range) | 8.6 (4.7-13.0) | 8.7 (5.8-13.0) | 8.0 (4.7-9.8) | 0.046* |
| Median CD34+ cell counts,  ×10^6^/kg (range) | 2.3 (0.7-7.3) | 2.3 (0.7-7.3) | 2.3 (1.4-6.0) | 0.631 |
| Neutrophil engraftment, n (%) | 80 (100.0) | 71 (100.0) | 9 (100.0) | 1.000 |
| Median time from HSCT to  neutrophil engraftment, d (range) | 12.5 (7-42) | 13 (7-42) | 12 (11-21) | 0.763 |
| Platelet engraftment, n (%) | 78 (97.5) | 69 (97.1) | 9 (100.0) | 0.612 |
| Median time from HSCT to platelet  engraftment, d (range) | 17 (9-220) | 17 (9-220) | 16 (12-102) | 0.970 |
| Severity of aGVHD, at the  beginning of the basiliximab  treatment, n (%) |  |  |  | 0.176 |
| < Grade Ⅲ | 73 (91.2) | 66 (93.0) | 7 (77.8) |  |
| ≥ Grade Ⅲ | 7 (8.8) | 5 (7.0) | 2 (22.2) |  |
| Median follow-up after HSCT, d  (range) | 1380 (330-2054) | 1367 (330-2054) | 1610 (1179-1984) | 0.142 |
| Median follow-up after basiliximab  treatment, d (range) | 1352 (312-2035) | 1349 (312-2035) | 1579 (1162-1906) | 0.140 |

IR, immune reconstitution; HID HSCT, haploidentical donor hematopoietic stem cell transplantation; HCT-CI, hematopoietic cell transplantation–specific comorbidity index; aGVHD, acute graft-versus-host disease. * means *P* < 0.05. ^#^Among a total of 327 patients achieving ORR, 80 were included for IR analysis at 9 months after HSCT, and the other 247 patients were excluded for the following situation: 1) 6 suffered aGVHD recurrence; 2) 63 had serious infections; 3) 7 had DLI; 4) 3 received second HSCT; 5) 64 had serious cGVHD; 6) 17 relapsed; 7) 5 suffered NRM; 8) 82 lacked of IR data.

**Table S4. Clinical characteristics of patients included for IR analysis at 12 months after HID HSCT (n=92)**

| **Characteristics** | Basiliximab any dose  (n=92^#^) | Basiliximab < 5 doses  (n=82) | Basiliximab ≥ 5 doses (n=10) | *P* value |
| --- | --- | --- | --- | --- |
| Age at HID HSCT, n (%) |  |  |  | 0.965 |
| <18 years | 50 (54.3) | 44 (53.7) | 6 (60.0) |  |
| ≥18 years | 42 (45.7) | 38 (46.3) | 4 (40.0) |  |
| Sex, n (%) |  |  |  | 1.000 |
| Male | 52 (56.5) | 46 (56.1) | 6 (60.0) |  |
| Female | 40 (43.5) | 36 (43.9) | 4 (40.0) |  |
| Underlying disease, n (%) |  |  |  | 0.148 |
| Acute leukemia | 56 (60.9) | 52 (63.4) | 4 (40.0) |  |
| Myelodysplastic syndrome | 8 (8.7) | 7 (8.5) | 1 (10.0) |  |
| Chronic myeloid leukemia | 1 (1.1) | 1 (1.2) | 0 (0.0) |  |
| Severe aplastic anemia | 24 (26.1) | 20 (24.4) | 4 (40.0) |  |
| Non-Hodgkin's lymphoma | 2 (2.2) | 2 (2.4) | 0 (0.0) |  |
| Multiple myeloma | 0 (0.0) | 0 (0.0) | 0 (0.0) |  |
| Myeloproliferative neoplasms | 0 (0.0) | 0 (0.0) | 0 (0.0) |  |
| Others | 1 (1.1) | 0 (0.0) | 1 (10.0) |  |
| HCT-CI score, n (%) |  |  |  | 0.157 |
| Low risk | 77 (83.7) | 67 (81.7) | 10 (100.0) |  |
| Intermediate risk | 8 (8.7) | 8 (9.8) | 0 (0.0) |  |
| High risk | 7 (7.6) | 7 (8.5) | 0 (0.0) |  |
| Donor–recipient relationship, n (%) |  |  |  | 0.376 |
| Father–child | 63 (68.5) | 56 (68.3) | 7 (70.0) |  |
| Mother–child | 6 (6.5) | 4 (4.9) | 2 (20.0) |  |
| Sibling–sibling | 12 (13.0) | 11 (13.4) | 1(10.0) |  |
| Child–parent | 9 (9.8) | 9 (11.0) | 0 (0.0) |  |
| Collateral related donor | 2 (2.2) | 2 (2.4) | 0 (0.0) |  |
| Donor-recipient sex matched, n (%) |  |  |  | 0.952 |
| Male to male | 42 (45.7) | 37 (45.1) | 5 (50.0) |  |
| Male to female | 33 (35.9) | 30 (36.6) | 3 (30.0) |  |
| Female to male | 10 (10.8) | 9 (11.0) | 1 (10.0) |  |
| Female to female | 7 (7.6) | 6 (7.3) | 1 (10.0) |  |
| Graft type, n (%) |  |  |  | 1.000 |
| Peripheral blood | 0 (0.0) | 0 (0.0) | 0 (0.0) |  |
| Bone marrow and peripheral blood | 92 (100.0) | 82 (100.0) | 10 (100.0) |  |
| Median mononuclear cell counts,  ×10^8^/kg (range) | 8.7 (5.8-14.1) | 8.7 (5.8-13.5) | 8.3 (7.2-14.1) | 0.880 |
| Median CD34+ cell counts,  ×10^6^/kg (range) | 2.3 (0.4-5.9) | 2.3 (0.4-5.9) | 2.1 (1.4-5.3) | 0.831 |
| Neutrophil engraftment, n (%) | 92 (100.0) | 82 (100.0) | 10 (100.0) | 1.000 |
| Median time from HSCT to  neutrophil engraftment, d (range) | 13 (7-42) | 12.5 (7-42) | 13 (11-21) | 0.338 |
| Platelet engraftment, n (%) | 90 (97.8) | 80 (97.5) | 10 (100.0) | 0.619 |
| Median time from HSCT to platelet  engraftment, d (range) | 17 (6-220) | 17 (6-220) | 18 (10-102) | 0.602 |
| Severity of aGVHD, at the  beginning of the basiliximab  treatment, n (%) |  |  |  | 0.039* |
| < Grade Ⅲ | 84 (91.3) | 77 (93.9) | 7 (70.0) |  |
| ≥ Grade Ⅲ | 8 (8.7) | 5 (6.1) | 3 (30.0) |  |
| Median follow-up after HSCT, d  (range) | 1432 (808-2054) | 1432 (808-2054) | 1482 (1025-1984) | 0.985 |
| Median follow-up after basiliximab  treatment, d (range) | 1400.5 (768-2035) | 1400.5 (768-2035) | 1456 (993-1906) | 0.970 |

IR, immune reconstitution; HID HSCT, haploidentical donor hematopoietic stem cell transplantation; HCT-CI, hematopoietic cell transplantation–specific comorbidity index; aGVHD, acute graft-versus-host disease. * means *P* < 0.05. ^#^Among a total of 327 patients achieving ORR, 92 were included for IR analysis at 12 months after HSCT, and the other 235 patients were excluded for the following situation: 1) 6 suffered aGVHD recurrence; 2) 70 had serious infections; 3) 8 had DLI; 4) 3 received second HSCT; 5) 65 had serious cGVHD; 6) 20 relapsed; 7) 5 suffered NRM; 8) 58 lacked of IR data.

**Table S5. Infections after 3 months following HID HSCT**

| **Types of infection** | ***n* (%)** | **Cured/NRM** |
| --- | --- | --- |
| **Viral infection** | 52 (29.1) | 45/7 |
| Cytomegalovirus | 14 (7.8) | 12/2 |
| Cytomegalovirus DNAemia | 9 (5.0) | 9/0 |
| Cytomegalovirus disease | 5 (2.8) | 3/2 |
| Epstein-Barr virus | 2 (1.2) | 1/1 |
| Epstein-Barr virus DNAemia | 1 (0.6) | 1/0 |
| Epstein-Barr virus related PTLD | 1 (0.6) | 0/1 |
| Herpes simplex virus | 7 (3.9) | 7/0 |
| Varicella zoster virus | 7 (3.9) | 7/0 |
| Influenza virus | 4 (2.2) | 3/1 |
| Respiratory syncytial virus | 2 (1.1) | 2/0 |
| Other virus infections | 16 (8.9) | 13/3 |
| **Serious bacterial infection** | 21 (11.7) | 19/2 |
| Pneumonia | 18 (10.1) | 16/2 |
| Gastrointestinal tract | 1 (0.6) | 1/0 |
| Skin | 1 (0.6) | 1/0 |
| Marrow | 1 (0.6) | 1/0 |
| **Invasive fungal infection** | 8 (4.5) | 6/2 |
| Pneumonia | 8 (4.5) | 6/2 |
| **Any infection** | 64 (35.8) | 57/7 |

HID HSCT, Haploidentical donor hematopoietic stem cell transplantation; NRM, non-relapse mortality; PTLD, Post-transplant lymphoproliferative disorders.

**Table S6. Infections after 6 months following HID HSCT**

| **Types of infection** | ***n* (%)** | **Cured/NRM** |
| --- | --- | --- |
| **Viral infection** | 20 (16.1) | 19/1 |
| Epstein-Barr virus | 1 (0.8) | 1/0 |
| Epstein-Barr virus related PTLD | 1 (0.8) | 1/0 |
| Herpes simplex virus | 4 (3.2) | 4/0 |
| Varicella zoster virus | 6 (4.8) | 6/0 |
| Influenza virus | 1 (0.8) | 1/0 |
| Respiratory syncytial virus | 2 (1.6) | 2/0 |
| Other virus infections | 6 (4.8) | 5/1 |
| **Serious bacterial infection** | 11 (8.9) | 9/2 |
| Pneumonia | 10 (8.1) | 8/2 |
| Marrow | 1 (0.8) | 1/0 |
| **Invasive fungal infection** | 2 (1.6) | 2/0 |
| Pneumonia | 2 (1.6) | 2/0 |
| **Any infection** | 28 (22.6) | 26/2 |

HID HSCT, haploidentical donor hematopoietic stem cell transplantation; NRM, non-relapse mortality;

PTLD, post-transplant lymphoproliferative disorders.

**Table S7. Infections after 12 months following HID HSCT**

| **Types of infection** | ***n* (%)** | **Cured/NRM** |
| --- | --- | --- |
| **Viral infection** | 9 (9.8) | 9/0 |
| Epstein-Barr virus | 2 (2.2) | 2/0 |
| Epstein-Barr virus DNAemia | 1 (1.1) | 1/0 |
| Epstein-Barr virus related PTLD | 1 (1.1) | 1/0 |
| Herpes simplex virus | 1 (1.1) | 1/0 |
| Varicella zoster virus | 2 (2.2) | 2/0 |
| Influenza virus | 1 (1.1) | 1/0 |
| Respiratory syncytial virus | 2 (2.2) | 2/0 |
| Other virus infections | 1 (1.1) | 1/0 |
| **Serious bacterial infection** | 1 (1.1) | 1/0 |
| Pneumonia | 1 (1.1) | 1/0 |
| **Invasive fungal infection** | 0 (0.0) | 0/0 |
| **Any infection** | 9 (9.8) | 9/0 |

HID HSCT, haploidentical donor hematopoietic stem cell transplantation; NRM, non-relapse mortality;

PTLD, post-transplant lymphoproliferative disorders.

**Table S8. Infections between patients with good and poor IR at 3 months after HID HSCT**

|  | ^#^Good IR (n=81) | Poor IR (n=98) | *P* value |
| --- | --- | --- | --- |
| Viral infection | 16.0% | 39.8% | <0.001** |
| Serious bacterial infection | 4.9% | 17.3% | 0.010* |
| Invasive fungal infection | 3.7% | 5.1% | 0.731 |
| Any infection | 21.0% | 48.0% | <0.001** |

IR, immune reconstitution; HID HSCT, haploidentical donor hematopoietic stem cell transplantation. **P* < 0.05, ***P* < 0.01.

^#^Good IR was defined as at least 3 types of major immune cells (i.e., monocytes, lymphocytes, CD19+ B cells, CD3+ T cells, CD4+ T cells, and CD8+ T cells) meeting or exceeding the median value of event-free HID HSCT cohort.

**Table S9. Infections between patients with good and poor IR at 6 months after HID HSCT**

|  | ^#^Good IR (n=76) | Poor IR (n=48) | *P* value |
| --- | --- | --- | --- |
| Viral infection | 17.1% | 14.6% | 0.710 |
| Serious bacterial infection | 5.3% | 14.6% | 0.105 |
| Invasive fungal infection | 0.0% | 4.2% | 0.148 |
| Any infection | 18.4% | 29.2% | 0.163 |

IR, immune reconstitution; HID HSCT, haploidentical donor hematopoietic stem cell transplantation.

^#^Good IR was defined as at least 3 types of major immune cells (i.e., monocytes, lymphocytes, CD19+ B cells, CD3+ T cells, CD4+ T cells, and CD8+ T cells) meeting or exceeding the median value of event-free HID HSCT cohort.

**Table S10. Infections between patients with good and poor IR at 12 months after HID HSCT**

|  | ^#^Good IR (n=66) | Poor IR (n=26) | *P* value |
| --- | --- | --- | --- |
| Viral infection | 10.6% | 7.7% | 1.000 |
| Serious bacterial infection | 1.5% | 0.0% | 1.000 |
| Invasive fungal infection | 0.0% | 0.0% | 1.000 |
| Any infection | 10.6% | 7.7% | 1.000 |

IR, immune reconstitution; HID HSCT, haploidentical donor hematopoietic stem cell transplantation.

^#^Good IR was defined as at least 3 types of major immune cells (i.e., monocytes, lymphocytes, CD19+ B cells, CD3+ T cells, CD4+ T cells, and CD8+ T cells) meeting or exceeding the median value of event-free HID HSCT cohort.

**Table S11. Clinical outcomes between patients with good and poor IR at 3 months after HID HSCT**

|  | ^#^Good IR (n=81) | Poor IR (n=98) | *P* value |
| --- | --- | --- | --- |
| cGVHD | 66.7% | 69.4% | 0.697 |
| NRM | 3.7% | 8.2% | 0.214 |
| DFS | 85.1% | 76.5% | 0.145 |
| OS | 88.7% | 83.7% | 0.203 |

IR, immune reconstitution; HID HSCT, haploidentical donor hematopoietic stem cell transplantation; cGVHD, chronic graft-versus-host disease; NRM, non-relapse mortality; DFS, disease-free survival; OS, overall survival.

^#^Good IR was defined as at least 3 types of major immune cells (i.e., monocytes, lymphocytes, CD19+ B cells, CD3+ T cells, CD4+ T cells, and CD8+ T cells) meeting or exceeding the median value of event-free HID HSCT cohort.

**Table S12. Clinical outcomes between patients with good and poor IR at 6 months after HID HSCT**

|  | ^#^Good IR (n=76) | Poor IR (n=48) | *P* value |
| --- | --- | --- | --- |
| cGVHD | 22.4% | 31.3% | 0.271 |
| NRM | 1.3% | 4.2% | 0.316 |
| DFS | 90.8% | 87.5% | 0.544 |
| OS | 92.6% | 91.7% | 0.512 |

IR, immune reconstitution; HID HSCT, haploidentical donor hematopoietic stem cell transplantation; cGVHD, chronic graft-versus-host disease; NRM, non-relapse mortality; DFS, disease-free survival; OS, overall survival.

^#^Good IR was defined as at least 3 types of major immune cells (i.e., monocytes, lymphocytes, CD19+ B cells, CD3+ T cells, CD4+ T cells, and CD8+ T cells) meeting or exceeding the median value of event-free HID HSCT cohort.

**Table S13. Clinical outcomes between patients with good and poor IR at 12 months after HID HSCT**

|  | ^#^Good IR (n=66) | Poor IR (n=26) | *P* value |
| --- | --- | --- | --- |
| cGVHD | 3.0% | 0.0% | 1.000 |
| NRM | 0.0% | 0.0% | 1.000 |
| DFS | 95.5% | 96.5% | 0.888 |
| OS | 95.4% | 100.0% | 0.353 |

IR, immune reconstitution; HID HSCT, haploidentical donor hematopoietic stem cell transplantation; cGVHD, chronic graft-versus-host disease; NRM, non-relapse mortality; DFS, disease-free survival; OS, overall survival.

^#^Good IR was defined as at least 3 types of major immune cells (i.e., monocytes, lymphocytes, CD19+ B cells, CD3+ T cells, CD4+ T cells, and CD8+ T cells) meeting or exceeding the median value of event-free HID HSCT cohort.

**Table S14. Infections between patients with good and poor IgA IR at 3 months after HID HSCT**

|  | ^#^Good IR (n=5) | Poor IR (n=174) | *P* value |
| --- | --- | --- | --- |
| Viral infection | 0.0% | 29.9% | 0.323 |
| Serious bacterial infection | 0.0% | 12.1% | 1.000 |
| Invasive fungal infection | 0.0% | 4.6% | 1.000 |
| Any infection | 0.0% | 36.8% | 0.162 |

IR, immune reconstitution; HID HSCT, haploidentical donor hematopoietic stem cell transplantation.

^#^Good IgA IR was defined as IgA meeting or exceeding the lower normal value.

**Table S15. Infections between patients with good and poor IgG IR at 3 months after HID HSCT**

|  | ^#^Good IR (n=115) | Poor IR (n=64) | *P* value |
| --- | --- | --- | --- |
| Viral infection | 28.7% | 29.7% | 0.889 |
| Serious bacterial infection | 13.0% | 9.4% | 0.465 |
| Invasive fungal infection | 5.2% | 3.1% | 0.713 |
| Any infection | 33.9% | 39.1% | 0.491 |

IR, immune reconstitution; HID HSCT, haploidentical donor hematopoietic stem cell transplantation.

^#^Good IgG IR was defined as IgG meeting or exceeding the lower normal value.

**Table S16. Infections between patients with good and poor IgM IR at 3 months after HID HSCT**

|  | ^#^Good IR (n=71) | Poor IR (n=108) | *P* value |
| --- | --- | --- | --- |
| Viral infection | 22.5% | 33.3% | 0.120 |
| Serious bacterial infection | 9.9% | 13.0% | 0.528 |
| Invasive fungal infection | 4.2% | 4.6% | 1.000 |
| Any infection | 26.8% | 41.7% | 0.042* |

IR, immune reconstitution; HID HSCT, haploidentical donor hematopoietic stem cell transplantation. **P* < 0.05.

^#^Good IgM IR was defined as IgM meeting or exceeding the lower normal value.

**Table S17. Infections between patients with good and poor IgA IR at 6 months after HID HSCT**

|  | ^#^Good IR (n=16) | Poor IR (n=108) | *P* value |
| --- | --- | --- | --- |
| Viral infection | 6.3% | 17.6% | 0.465 |
| Serious bacterial infection | 6.3% | 9.3% | 1.000 |
| Invasive fungal infection | 0.0% | 1.9% | 1.000 |
| Any infection | 6.3% | 25.0% | 0.117 |

IR, immune reconstitution; HID HSCT, haploidentical donor hematopoietic stem cell transplantation.

^#^Good IgA IR was defined as IgA meeting or exceeding the lower normal value.

**Table S18. Infections between patients with good and poor IgG IR at 6 months after HID HSCT**

|  | ^#^Good IR (n=88) | Poor IR (n=36) | *P* value |
| --- | --- | --- | --- |
| Viral infection | 14.8% | 19.4% | 0.521 |
| Serious bacterial infection | 9.1% | 8.3% | 1.000 |
| Invasive fungal infection | 1.1% | 2.8% | 0.498 |
| Any infection | 21.6% | 25.0% | 0.680 |

IR, immune reconstitution; HID HSCT, haploidentical donor hematopoietic stem cell transplantation.

^#^Good IgG IR was defined as IgG meeting or exceeding the lower normal value.

**Table S19. Infections between patients with good and poor IgM IR at 6 months after HID HSCT**

|  | ^#^Good IR (n=89) | Poor IR (n=35) | *P* value |
| --- | --- | --- | --- |
| Viral infection | 18.0% | 11.4% | 0.372 |
| Serious bacterial infection | 7.9% | 11.4% | 0.503 |
| Invasive fungal infection | 2.2% | 0.0% | 1.000 |
| Any infection | 24.7% | 17.1% | 0.364 |

IR, immune reconstitution; HID HSCT, haploidentical donor hematopoietic stem cell transplantation.

^#^Good IgM IR was defined as IgM meeting or exceeding the lower normal value.

**Table S20. Infections between patients with good and poor IgA IR at 12 months after HID HSCT**

|  | ^#^Good IR (n=29) | Poor IR (n=63) | *P* value |
| --- | --- | --- | --- |
| Viral infection | 6.9% | 11.1% | 0.714 |
| Serious bacterial infection | 0.0% | 1.6% | 1.000 |
| Invasive fungal infection | 0.0% | 0.0% | 1.000 |
| Any infection | 6.9% | 11.1% | 0.714 |

IR, immune reconstitution; HID HSCT, haploidentical donor hematopoietic stem cell transplantation.

^#^Good IgA IR was defined as IgA meeting or exceeding the lower normal value.

**Table S21. Infections between patients with good and poor IgG IR at 12 months after HID HSCT**

|  | ^#^Good IR (n=78) | Poor IR (n=14) | *P* value |
| --- | --- | --- | --- |
| Viral infection | 10.3% | 7.1% | 1.000 |
| Serious bacterial infection | 1.3% | 0.0% | 1.000 |
| Invasive fungal infection | 0.0% | 0.0% | 1.000 |
| Any infection | 10.3% | 7.1% | 1.000 |

IR, immune reconstitution; HID HSCT, haploidentical donor hematopoietic stem cell transplantation.

^#^Good IgG IR was defined as IgG meeting or exceeding the lower normal value.

**Table S22. Infections between patients with good and poor IgM IR at 12 months after HID HSCT**

|  | ^#^Good IR (n=77) | Poor IR (n=15) | *P* value |
| --- | --- | --- | --- |
| Viral infection | 11.7% | 0.0% | 0.346 |
| Serious bacterial infection | 1.3% | 0.0% | 1.000 |
| Invasive fungal infection | 0.0% | 0.0% | 1.000 |
| Any infection | 11.7% | 0.0% | 0.346 |

IR, immune reconstitution; HID HSCT, haploidentical donor hematopoietic stem cell transplantation.

^#^Good IgM IR was defined as IgM meeting or exceeding the lower normal value.

**Table S23. Immune reconstitution in SR–aGVHD patients who had immune reconstitution data at all 4 monitoring points (median (25th–75th))**

|  | Basiliximab any dose (n=36) | Basiliximab < 5 doses (n=32) | Basiliximab ≥ 5 doses (n=4) | *P* value |
| --- | --- | --- | --- | --- |
| 3m |  |  |  |  |
| Monocyte | 383.10 (244.51–525.00) | 414.18 (244.51–527.48) | 289.11 (160.76–471.00) | 0.450 |
| Lymphocyte | 1179.88 (619.89–2001.20) | 1179.88 (619.89–1988.48) | 1362.20 (417.73–2052.42) | 0.981 |
| CD19+ B cell | 12.47 (3.02–35.88) | 11.60 (1.98–35.88) | 13.24 (5.74–53.89) | 0.827 |
| CD3+ T cell | 773.92 (498.20–1598.78) | 764.95 (498.20–1626.56) | 1134.30 (355.48–1597.90) | 0.903 |
| CD4+ T cell | 108.99 (58.29–249.72) | 112.85 (58.29–280.40) | 77.77 (27.05–139.05) | 0.340 |
| CD8+ T cell | 629.04 (370.18–1210.26) | 585.12 (370.18–1184.60) | 955.57 (324.71–1358.32) | 0.576 |
| CD4+ naïve T cell | 2.63 (1.00-5.75) | 2.63 (1.05-5.75) | 2.20 (0.28-6.62) | 0.680 |
| CD4+ memory T cell | 104.02 (50.10-230.58) | 106.91 (50.10-262.35) | 75.40 (25.90-123.32) | 0.393 |
| CD4+CD25+ T cell | 1.22 (0.00–13.54) | 1.22 (0.00–14.34) | 4.72 (0.05–11.97) | 0.865 |
| CD4+CD28+ T cell | 81.92 (52.42–115.71) | 87.39 (52.42–129.15) | 72.38 (26.35–79.60) | 0.269 |
| CD8+CD28+ T cell | 107.92 (60.73–230.76) | 107.92 (60.73–255.53) | 131.60 (32.86–198.10) | 0.865 |
| CD4–CD8– T cell | 36.43 (13.17–96.45) | 39.56 (13.87–98.64) | 31.61 (4.22–75.66) | 0.476 |
| IgA (G/L) | 0.40 (0.17–0.51) | 0.37 (0.17–0.51) | 0.42 (0.22–0.53) | 0.790 |
| IgG (G/L) | 9.1 (6.2–11.9) | 9.2 (6.3–11.9) | 4.5 (4.3–14.3) | 0.128 |
| IgM (G/L) | 0.379 (0.207–0.645) | 0.428 (0.207–0.674) | 0.303 (0.116–0.380) | 0.208 |
| 6m |  |  |  |  |
| Monocyte | 440.03 (383.00–537.14) | 439.52 (383.00–537.14) | 495.90 (260.51–566.65) | 0.716 |
| Lymphocyte | 1806.35 (1016.72–2781.59) | 1806.35 (1016.72–2876.90) | 1725.12 (939.17–2509.84) | 0.790 |
| CD19+ B cell | 40.02 (23.00–134.80) | 46.48 (23.00–134.80) | 35.43 (18.25–268.81) | 0.752 |
| CD3+ T cell | 1433.10 (907.60–2186.97) | 1433.10 (907.60–2326.49) | 1626.09 (737.89–2093.34) | 0.942 |
| CD4+ T cell | 189.69 (117.56–373.72) | 189.69 (119.19–386.05) | 163.46 (61.37–200.51) | 0.315 |
| CD8+ T cell | 1057.79 (701.80–1791.64) | 1046.01 (701.80–1791.64) | 1319.39 (631.93–1840.10) | 0.752 |
| CD4+ naïve T cell | 8.76 (3.55-30.00) | 8.76 (4.05-30.00) | 10.17 (1.07-30.46) | 0.645 |
| CD4+ memory T cell | 156.90 (86.62-298.70) | 169.43 (86.62-312.89) | 140.03 (59.61-159.79) | 0.248 |
| CD4+CD25+ T cell | 22.79 (12.28–43.03) | 25.64 (12.28–49.37) | 20.41 (10.29–32.91) | 0.576 |
| CD4+CD28+ T cell | 138.16 (73.13–201.42) | 141.70 (78.90–217.01) | 107.81 (48.32–175.58) | 0.393 |
| CD8+CD28+ T cell | 144.21 (91.56–208.88) | 146.16 (83.18–208.88) | 115.66 (103.93–219.35) | 0.827 |
| CD4–CD8– T cell | 79.97 (41.85–125.11) | 80.40 (42.61–125.93) | 60.55 (13.86–114.34) | 0.366 |
| IgA (G/L) | 0.38 (0.25–0.58) | 0.38 (0.25–0.58) | 0.47 (0.27–0.59) | 0.680 |
| IgG (G/L) | 9.7 (6.9–12.3) | 9.5 (6.9–11.8) | 11.5 (7.0–14.4) | 0.511 |
| IgM (G/L) | 0.705 (0.432–0.978) | 0.705 (0.427–0.978) | 0.669 (0.469–1.982) | 0.865 |
| 9m |  |  |  |  |
| Monocyte | 494.13 (337.76–632.04) | 496.32 (337.76–632.63) | 454.44 (276.50–597.65) | 0.680 |
| Lymphocyte | 2528.67 (1677.29–3621.41) | 2528.57 (1677.29–3734.18) | 2370.90（1694.50–3347.61) | 0.790 |
| CD19+ B cell | 173.51 (61.85–322.80) | 207.79 (61.85–322.80) | 104.71（55.19–582.36) | 0.903 |
| CD3+ T cell | 2004.63 (1350.97–3275.17) | 1845.19 (1298.36–3305.66) | 2284.44（1708.40–2956.35) | 0.752 |
| CD4+ T cell | 327.25 (176.06–501.64) | 327.25 (177.06–583.01) | 266.40（152.28–462.96) | 0.610 |
| CD8+ T cell | 1555.75 (948.76–2411.76) | 1476.87 (869.29–2454.20) | 2031.49（1337.18–2386.50) | 0.543 |
| CD4+ naïve T cell | 23.99 (6.14-100.39) | 23.99 (6.14-93.58) | 60.84 (5.79-211.55) | 0.865 |
| CD4+ memory T cell | 239.89 (154.16-318.03) | 249.05 (157.83-332.61) | 180.56 (143.93-233.75) | 0.248 |
| CD4+CD25+ T cell | 22.28 (13.79–43.25) | 22.28 (13.33–43.25) | 26.94（15.29–47.27) | 1.000 |
| CD4+CD28+ T cell | 229.36 (135.55–406.37) | 231.16 (135.55–406.37) | 195.83（113.41–397.21) | 0.716 |
| CD8+CD28+ T cell | 158.46 (107.94–277.52) | 182.60 (102.50–289.73) | 149.72（129.36–188.90) | 0.610 |
| CD4–CD8– T cell | 101.13 (73.46–153.90) | 101.13 (74.52–151.72) | 89.81（31.13–182.63) | 0.790 |
| IgA (G/L) | 0.55 (0.31–0.76) | 0.53 (0.31–0.75) | 0.79 (0.40–0.94) | 0.340 |
| IgG (G/L) | 10.9 (8.5–13.1) | 10.9 (8.5–13.1) | 11.5 (8.6–15.0) | 0.865 |
| IgM (G/L) | 0.904 (0.566–1.305) | 0.840 (0.516–1.258) | 1.205 (0.968–2.888) | 0.115 |
| 12m |  |  |  |  |
| Monocyte | 427.97 (343.61–549.22) | 412.24 (343.61–538.32) | 565.98 (376.71–645.42) | 0.480 |
| Lymphocyte | 2794.53 (1908.52–3968.43) | 2900.63 (1991.60–3968.43) | 2394.50 (1907.56–4530.33) | 0.827 |
| CD19+ B cell | 307.58 (149.38–468.79) | 307.58 (151.50–468.79) | 247.85 (77.62–801.53) | 0.865 |
| CD3+ T cell | 2038.03 (1425.09–2975.98) | 2038.03 (1425.09–2975.98) | 2030.32 (1478.67–3534.09) | 1.000 |
| CD4+ T cell | 450.58 (242.11–683.82) | 450.58 (242.11–683.82) | 435.41 (183.83–686.29) | 0.790 |
| CD8+ T cell | 1344.50 (1053.59–2269.31) | 1344.50 (1039.35–2269.31) | 1579.30 (1193.14–2706.65) | 0.543 |
| CD4+ naïve T cell | 77.83 (18.48-256.56) | 75.63 (18.48-256.56) | 146.53 (34.31-352.23) | 0.865 |
| CD4+ memory T cell | 226.30 (159.54-342.81) | 222.37 (159.54-366.15) | 237.56 (143.85-265.53) | 0.716 |
| CD4+CD25+ T cell | 30.96 (17.19–66.19) | 34.97 (17.47–67.26) | 20.18 (7.15–56.84) | 0.248 |
| CD4+CD28+ T cell | 321.21 (151.82–564.29) | 319.88 (151.82–564.29) | 332.89 (136.14–589.03) | 0.903 |
| CD8+CD28+ T cell | 200.31 (138.70–366.46) | 206.35 (143.70–378.09) | 151.31 (109.76–296.05) | 0.269 |
| CD4–CD8– T cell | 89.71 (58.39–143.84) | 92.83 (61.44–143.84) | 64.82 (40.90–166.94) | 0.450 |
| IgA (G/L) | 0.61 (0.38–0.83) | 0.57 (0.38–0.83) | 0.90 (0.46–1.05) | 0.292 |
| IgG (G/L) | 11.8 (9.1–14.8) | 11.8 (9.1–14.8) | 11.8 (8.7–15.2) | 0.981 |
| IgM (G/L) | 0.914 (0.554–1.178) | 0.865 (0.507–1.165) | 1.130 (0.863–1.900) | 0.157 |

SR-aGVHD, steroid-refractory acute graft-versus-host disease.

**Table S24. Immune reconstitution between SR-aGVHD patients who had immune reconstitution data at all 4 monitoring points and event-free HID HSCT recipients (median (25th–75th))**

|  | Basiliximab any dose (n=36) | Event-free HID HSCT (n=85) | *P* |
| --- | --- | --- | --- |
| 3m |  |  |  |
| Monocyte | 383.10 (244.51–525.00) | 450.45 (311.70–580.11) | 0.076 |
| Lymphocyte | 1179.88 (619.89–2001.20) | 1238.36 (852.24–1964.93) | 0.472 |
| CD19+ B cell | 12.47 (3.02–35.88) | 11.26 (4.58–26.52) | 0.792 |
| CD3+ T cell | 773.92 (498.20–1598.78) | 834.83 (524.55–1341.85) | 0.708 |
| CD4+ T cell | 108.99 (58.29–249.72) | 109.71 (63.11–190.08) | 0.883 |
| CD8+ T cell | 629.04 (370.18–1210.26) | 684.00 (392.11–1098.09) | 0.658 |
| CD4+CD25+ T cell | 1.22 (0.00–13.54) | 23.29 (13.32–42.70) | <0.001** |
| CD4+CD28+ T cell | 81.92 (52.42– 115.71) | 11.62 (6.14–24.37) | <0.001** |
| CD8+CD28+ T cell | 107.92 (60.73–230.76) | 157.88 (88.65–223.41) | 0.229 |
| CD4–CD8– T cell | 36.43 (13.17–96.45) | 30.41 (15.77–59.32) | 0.633 |
| 6m |  |  |  |
| Monocyte | 440.03 (383.00–537.14) | 460.35 (310.28–579.55) | 0.683 |
| Lymphocyte | 1806.35 (1016.72–2871.59) | 1669.80 (1149.33–2449.45) | 0.477 |
| CD19+ B cell | 40.02 (23.00–134.80) | 69.84 (26.26–162.78) | 0.334 |
| CD3+ T cell | 1433.10 (907.60–2186.97) | 1194.15 (781.83–1879.41) | 0.200 |
| CD4+ T cell | 189.69 (117.56–373.72) | 173.43 (112.29–285.73) | 0.437 |
| CD8+ T cell | 1057.79 (701.80–1791.64) | 945.63 (596.17–1426.72) | 0.292 |
| CD4+CD25+ T cell | 22.79 (12.28–43.03) | 48.58 (33.89–86.98) | <0.001** |
| CD4+CD28+ T cell | 138.16 (73.13–201.42) | 22.38 (11.98–41.31) | <0.001** |
| CD8+CD28+ T cell | 144.21 (91.56–208.88) | 138.33 (92.38–248.66) | 0.892 |
| CD4–CD8– T cell | 79.97 (41.85–125.11) | 41.19 (24.43–91.84) | 0.009** |
| 12m |  |  |  |
| Monocyte | 427.97 (343.61–549.22) | 499.38 (390.74–615.03) | 0.123 |
| Lymphocyte | 2794.53 (1908.52–3968.43) | 2232.38 (1550.55–2856.45) | 0.009** |
| CD19+ B cell | 307.58 (149.38–468.79) | 242.95 (118.14–371.36) | 0.170 |
| CD3+ T cell | 2038.03 (1425.09–2975.98) | 1561.06 (1162.98–2408.78) | 0.024* |
| CD4+ T cell | 450.58 (242.11– 683.82) | 305.86 (230.81–438.35) | 0.025* |
| CD8+ T cell | 1344.50 (1053.59–2269.31) | 1035.30 (809.95–1761.59) | 0.050 |
| CD4+CD25+ T cell | 30.96 (17.19– 66.19) | 91.28 (57.33–119.26) | <0.001** |
| CD4+CD28+ T cell | 321.21 (151.82–564.29) | 47.45 (27.50–80.94) | <0.001** |
| CD8+CD28+ T cell | 200.31(138.70–366.46) | 175.65 (107.79–265.33) | 0.076 |
| CD4–CD8– T cell | 89.71(58.39–143.84) | 189.99 (123.72–368.34) | <0.001** |

SR-aGVHD, steroid-refractory acute graft-versus-host disease; HID HSCT, haploidentical donor hematopoietic stem cell transplantation. **P* < 0.05, ***P* < 0.01.

**Table S25. Immune reconstitution between SR-aGVHD patients and event-free HID HSCT recipients at 1 and 2 months after HSCT (median (25th–75th))**

|  | Basiliximab any dose | Event-free HID | *P* value |
| --- | --- | --- | --- |
| IR at 1m  Monocyte  Lymphocyte  CD19+ B cell  CD3+ T cell  CD4+ T cell  CD8+ T cell  CD4+CD25+ T cell  CD4+CD28+ T cell  CD8+CD28+ T cell  CD4–CD8– T cell | (n=180^#^)  448.02 (259.47–729.59)  423.93 (230.29–749.33)  5.62 (1.97–11.77)  160.42 (79.31–332.29)  26.20 (12.88–46.32)  106.56 (52.89–257.99)  0.00 (0.00–1.36)  23.74 (11.52–45.03)  42.89 (19.93–108.03)  12.17 (5.17–31.56) | (n=85)  567.84 (339.63–914.39)  289.20 (129.58–414.16)  5.28 (2.54–11.82)  110.64 (57.62–250.11)  24.31 (9.27–69.34)  71.92 (35.10–148.97)  11.18 (2.84–21.80)  5.34 (1.33–14.87)  23.95 (10.99–65.94)  7.66 (2.50–15.26) | 0.009**  <0.001**  0.787  0.061  0.984  0.023*  <0.001**  <0.001**  0.003**  0.007** |
| IR at 2m  Monocyte  Lymphocyte  CD19+ B cell  CD3+ T cell  CD4+ T cell  CD8+ T cell  CD4+CD25+ T cell  CD4+CD28+ T cell  CD8+CD28+ T cell  CD4–CD8– T cell | (n=151)  339.60 (183.30–567.00)  790.92 (432.00–1351.27)  5.46 (2.31–9.90)  585.50 (231.70–1054.35)  53.24 (29.56–147.36)  449.68 (167.83–841.23)  0.00 (0.00–0.33)  45.54 (21.12–100.80)  84.79 (38.85–187.60)  27.30 (10.17–64.23) | (n=85)  400.50 (228.74–561.27)  848.70 (486.09–1451.15)  11.00 (4.06–27.05)  608.26 (350.89–1089.21)  93.12 (54.81–148.07)  493.93 (240.75–859.97)  19.07 (7.11–37.20)  12.03 (6.23–27.72)  126.50 (56.31–228.04)  24.41 (11.06–57.93) | 0.257  0.353  <0.001**  0.190  0.002**  0.284  <0.001**  <0.001**  0.031*  0.946 |

IR, immune reconstitution; HID HSCT, haploidentical donor hematopoietic stem cell transplantation; SR-aGVHD, steroid-refractory acute graft-versus-host disease. ^#^Among a total of 231 SR-aGVHD patients included in the analysis for IR, 180 and 151 patients had IR data available at 1 and 2 months following HSCT, respectively. **P* < 0.05, ***P* < 0.01.

**Table S26. The percentage of immune cell subset at 3, 6, 9, and 12 months after HID HSCT (median (25th–75th))**

|  | Basiliximab any dose (%) | Basiliximab < 5 doses (%) | Basiliximab ≥ 5 doses (%) |
| --- | --- | --- | --- |
| 3m | (n=179) | (n=142) | (n=37) |
| Monocyte | 9.80 (7.50–13.10) | 9.90 (7.43–12.55) | 8.90 (7.05–15.15) |
| Lymphocyte | 26.90 (16.80–41.80) | 27.00 (16.30–40.90) | 25.50 (18.05–49.40) |
| CD19+ B cell | 0.23 (0.08–0.53) | 0.21 (0.07–0.46) | 0.34 (0.12–0.72) |
| CD3+ T cell | 21.14 (12.34–34.20) | 19.61 (12.27–33.54) | 22.36 (12.56–38.12) |
| CD4+ T cell | 2.77 (1.54–4.71) | 2.53 (1.50–4.24) | 3.31 (1.89–5.77) |
| CD8+ T cell | 16.39 (9.7–26.67) | 16.27 (9.69–25.74) | 18.30 (9.96–29.61) |
| CD4+ naïve T cell  CD4+ memory T cell  CD4+CD25+ T cell | 0.06 (0.02–0.12)  2.44 (1.48–4.34)  0.01 (0.00–0.21) | 0.06 (0.02–0.11)  1.45 (2.31–4.11)  0.03 (0.00–0.23) | 0.06 (0.02–0.16)  2.78 (1.97–5.07)  0.00 (0.00–0.08) |
| CD4+CD28+ T cell | 2.10 (1.20–3.30) | 2.05 (1.09–3.22) | 2.52 (1.73–3.50) |
| CD8+CD28+ T cell | 3.26 (1.70–5.30) | 3.05 (1.54–5.23) | 3.79 (1.94–5.79) |
| CD4–CD8– T cell | 0.96 (0.41–2.26) | 0.93 (0.41–2.43) | 1.22 (0.41–2.22) |
| 6m | (n=124) | (n=104) | (n=20) |
| Monocyte | 8.95 (6.80–10.88) | 8.70 (6.30–10.48) | 10.75 (8.55–12.85) |
| Lymphocyte | 40.40 (31.45–52.90) | 39.80 (29.93–50.93) | 45.85 (32.18–56.43) |
| CD19+ B cell | 0.90 (0.34–2.55) | 0.92 (0.31–2.55) | 0.79 (0.37–3.12) |
| CD3+ T cell | 33.63 (23.34–43.28) | 33.63 (20.55–42.50) | 34.51 (25.10–46.36) |
| CD4+ T cell | 4.67 (2.91–6.99) | 4.72 (2.90–6.99) | 4.62 (2.98–6.91) |
| CD8+ T cell | 26.39 (16.39–33.03) | 26.23 (16.31–33.01) | 28.07 (16.39–34.65) |
| CD4+ naïve T cell  CD4+ memory T cell  CD4+CD25+ T cell | 0.16 (0.07–0.44)  4.05 (2.64–5.72)  0.45 (0.19–0.75) | 0.17 (0.07–0.44)  3.98 (2.62–5.99)  0.44 (0.19–0.77) | 0.13 (0.06–0.46)  4.19 (2.71–5.39)  0.46 (0.12–0.60) |
| CD4+CD28+ T cell | 3.11 (1.71–4.43) | 3.03 (1.63–4.54) | 3.22 (2.60–4.39) |
| CD8+CD28+ T cell | 3.58 (1.80–5.03) | 3.29 (1.69–4.90) | 4.91 (3.58–5.79) |
| CD4–CD8– T cell | 1.84 (1.01–2.72) | 1.86 (1.01–2.72) | 1.72 (0.96–3.22) |
| 9m | (n=80) | (n=71) | (n=9) |
| Monocyte | 7.50 (5.83–10.03) | 7.40 (5.80–11.10) | 7.70 (5.85–8.95) |
| Lymphocyte | 50.70 (35.63–57.15) | 51.40 (35.00–57.20) | 49.00 (38.20–56.15) |
| CD19+ B cell | 3.15 (1.06–6.12) | 3.23 (0.88–6.20) | 2.92 (1.24–5.42) |
| CD3+ T cell | 40.66 (31.33–49.14) | 40.60 (31.06–49.17) | 42.63 (38.82–49.40) |
| CD4+ T cell | 5.87 (3.91–8.79) | 5.70 (3.94–8.80) | 6.42 (3.46–8.07) |
| CD8+ T cell | 30.24 (22.74–39.41) | 29.70 (21.28–39.59) | 34.11 (27.52–39.73) |
| CD4+ naïve T cell  CD4+ memory T cell  CD4+CD25+ T cell | 0.49 (0.15–1.53)  4.35 (2.91–6.05)  0.50 (0.25–0.82) | 0.48 (0.14–1.52)  4.35 (2.89–6.42)  0.47 (0.24–0.84) | 0.81 (0.25–2.61)  3.36 (3.05–5.55)  0.72 (0.38–0.79) |
| CD4+CD28+ T cell | 3.73 (2.40–5.95) | 3.69 (2.38–5.93) | 5.62 (3.08–6.28) |
| CD8+CD28+ T cell | 3.40 (1.90–5.22) | 3.27 (1.73–5.03) | 3.76 (2.63–7.98) |
| CD4–CD8– T cell | 1.98 (1.28–2.71) | 2.00 (1.33–2.70) | 1.77 (0.98–3.25) |
| 12m | (n=92) | (n=82) | (n=10) |
| Monocyte | 7.75 (6.10–9.90) | 7.70 (6.10–9.90) | 8.55 (5.63–9.90) |
| Lymphocyte | 49.05 (38.83–61.53) | 49.05 (39.20–61.78) | 49.00 (34.02–56.48) |
| CD19+ B cell | 3.76 (2.02–7.30) | 4.05 (2.21–7.31) | 2.24 (1.57–6.89) |
| CD3+ T cell | 36.76 (27.28–47.40) | 37.06 (26.96–47.60) | 32.23 (27.80–48.47) |
| CD4+ T cell | 7.75 (5.58–11.29) | 7.75 (5.50–11.40) | 7.50 (5.37–9.67) |
| CD8+ T cell | 24.64 (18.24–33.03) | 25.03 (17.81–32.88) | 22.12 (17.86–35.45) |
| CD4+ naïve T cell  CD4+ memory T cell  CD4+CD25+ T cell | 1.49 (0.45–3.84)  5.05 (3.66–6.30)  0.71 (0.37–1.11) | 1.49 (0.48–3.74)  5.05 (3.65–6.46)  0.71 (0.37–1.11) | 1.65 (0.37–3.68)  4.72 (3.68–6.02)  0.71 (0.38–1.11) |
| CD4+CD28+ T cell | 5.50 (3.19–8.59) | 5.34 (3.15–8.64) | 5.58 (3.49–8.43) |
| CD8+CD28+ T cell | 3.71 (2.38–5.09) | 3.71 (2.36–5.16) | 3.44 (2.30–5.16) |
| CD4–CD8– T cell | 1.87 (1.20–2.82) | 1.89 (1.20–2.74) | 1.64 (0.76–3.56) |

The percentage = immune cell subset count/white blood cell count at the same monitoring point. HID HSCT, haploidentical donor hematopoietic stem cell transplantation.
